# Are there benefits from using bone-borne maxillary expansion instead of tooth-borne maxillary expansion? A systematic review with meta-analysis

**DOI:** 10.1186/s40510-019-0261-5

**Published:** 2019-02-25

**Authors:** Marietta Krüsi, Theodore Eliades, Spyridon N. Papageorgiou

**Affiliations:** Clinic of Orthodontics and Pediatric Dentistry, Center of Dental Medicine, University of Zurich, Plattenstrasse 11, CH-8032 Zurich, Switzerland

**Keywords:** Orthodontics, Maxillary expansion, Skeletal anchorage, Effectiveness, Adverse effects, Clinical trials, Systematic review, Meta-analysis

## Abstract

**Background:**

The aim of the current systematic review was to compare the clinical effects of bone-borne or hybrid tooth-bone-borne rapid maxillary expansion (RME) with conventional tooth-borne RME in the treatment of maxillary deficiency.

**Methods:**

Nine databases were searched up to September 2018 for randomized clinical trials comparing bone-borne or hybrid tooth-bone-borne RME to conventional tooth-borne RME in patients of any age or sex. After duplicate study selection, data extraction, and risk of bias assessment with the Cochrane tool, random effects meta-analyses of mean differences (MD) and their 95% confidence intervals (CIs) were performed, followed by assessment of the quality of evidence with GRADE.

**Results:**

A total of 12 papers on 6 unique trials with 264 patients (42.4% male; average age 12.3 years) were finally included. Limited evidence indicated that bone-borne RME was associated with greater suture opening at the first molar post-retention (1 trial; MD 2.0 mm; 95% CI 1.4 to 2.6 mm; moderate evidence quality) compared to tooth-borne RME, while no significant differences could be found regarding tooth inclination, nasal cavity width, and root resorption (very low to low evidence quality). Hybrid tooth-bone-borne RME was associated with less buccal tipping of the first premolar (2 trials; MD − 4.0°; 95% CI − 0.9 to − 7.1°; moderate evidence quality) and lower nasal airway resistance post-retention (1 trial; MD − 0.2 Pa s/cm^3^; 95% CI − 0.4 to 0 Pa s/cm^3^; moderate evidence quality) compared to tooth-borne RME, while no significant difference could be found regarding skeletal maxillary width, molar inclination, and analgesic use (low to moderate evidence quality). The main limitations affecting the validity of the present findings were (a) imprecision due to the inclusion of few trials with limited sample sizes that precluded robust detection of existing differences and (b) methodological issues of the included trials that could lead to bias.

**Conclusions:**

Limited evidence from randomized trials indicates that bone-borne or hybrid tooth-bone-borne RME might present advantages in terms of increased sutural opening, reduced tooth tipping, and lower nasal airway resistance compared to conventional tooth-borne RME. However, the limited number of existing studies and issues in their conduct or reporting preclude the drawing of definite conclusions.

**Review registration:**

PROSPERO (CRD42017079107).

**Electronic supplementary material:**

The online version of this article (10.1186/s40510-019-0261-5) contains supplementary material, which is available to authorized users.

## Introduction

Transverse maxillary deficiency is a malocclusion seen among adolescents or adults with prevalence over 8–10% [1, 2] and can manifest clinically as unilateral or bilateral crossbite, narrow nasal cavity, arch length discrepancy, and crowding [3, 4]. Additionally, some evidence indicates that posterior crossbites might be associated with temporomandibular disorders, including clicking and muscle tenderness [5]. Therefore, transverse maxillary deficiencies are usually treated on diagnosis to enable the settling of a harmonic occlusion, while avoiding any potential side effects.

In the treatment of transverse maxillary deficiencies, especially among adolescents, orthopedic expansion of the maxilla along the median palatal suture holds a prominent place [6]. This usually follows the protocol of a rapid maxillary expansion (RME), where the palatal expander is fixed on the maxillary posterior teeth. RME using tooth-borne expanders has been shown to be an effective alternative for the treatment of maxillary transverse deficiency [7] among adolescents, with treatment effects including an expansion of the maxillary arch (being mostly dental and less skeletal [8, 9]), widening of the nasal cavity [10], anterior movement of the maxilla [11] with a downward rotation [12], and a small spontaneous increase in mandibular arch width [9]. On the other side, tooth-anchored RME has also been associated with some adverse effects to the teeth and the surrounding tissues, including among others, buccal tooth tipping [11], reduced buccal bone thickness [13], marginal bone loss [13], bone fenestration [14], buccal gingival recessions [15], and root resorption [16].

In order to overcome these potential limitations and possibly enhance the skeletal effects of conventional tooth-borne RME, the use of an RME anchored completely or partly on skeletal anchorage devices was proposed [17], designated as bone-borne or hybrid (tooth-bone-borne) RME, respectively. The suggested benefits of such appliances include greater skeletal expansion of the maxilla and facial bones, reduced burden and adverse effects on the anchorage teeth, and improved stability of the results. These benefits come of course at the cost of increased invasiveness of the procedure and increased risk of wound infection [18].

Even though research on this field continues to increase, clinical evidence about the comparative performance of skeletally anchored RME has not been systematically and critically appraised. Therefore, the aim of the present systematic review was to compare the efficacy and adverse effects of partially/completely skeletally anchored RME versus conventional (tooth-borne) RME for the treatment of maxillary transverse deficiency based on evidence from randomized clinical trials.

## Material and methods

### Protocol, eligibility criteria, and registration

This review’s protocol was made a priori, registered in PROSPERO (CRD42017079107), and all post hoc changes were appropriately noted. This systematic review was conducted and reported according to Cochrane Handbook [19] and Preferred Reporting Items for Systematic Reviews and Meta-Analyses (PRISMA) statement [20], respectively.

Based on the Participants-Interventions-Comparisons-Outcome-Study design (PICOS) approach, we included randomized controlled clinical trials on human patients of any age or sex with transverse maxillary deficiency treated with bone-borne compared to tooth-borne maxillary expansion in terms of skeletal expansion as the primary outcome. Two discreet experimental interventions were considered eligible and compared with the conventional tooth-borne RME control: purely bone-borne RME, as well as hybrid tooth-bone-borne RME. Non-randomized studies, animal studies, in vitro studies, and studies, where RME was surgically assisted, were excluded.

### Information sources and literature search

The following nine electronic databases were systematically searched for this review: MEDLINE (via PubMed), Embase, The Cochrane Library (CDSR, CENTRAL, and DARE), Virtual Health Library (including Bibliography Brazilian Dentistry and LILACS), Scopus, ISI Web of Knowledge, and ClinicalTrials.gov (Additional file 1: Appendix 1). Manual searches were applied on the databases Directory of Open Access Journals (DOAJ), Digital Dissertations (via UMI Proquest), metaRegister of Controlled Trials, WHO trials search portal, and Google Scholar for additional trials as well as for the reference lists of the included studies. The entire search was made by one author (SNP) without any limitations from inception of each database up to September 29, 2018. Aside from filtering trials on humans, no other filters for language, publications year, and status were applied.

### Study selection and data collection

The identified studies from the literature search were sequentially screened by title, abstract, and full text by one author (MK) with subsequent duplicate independent checking against the eligibility criteria by another author (SNP), while conflicts were resolved by a third author (TE).

The same protocol was applied for the extraction of study characteristics (study design, setting, country, patient number, sex, age, appliances, treatment duration, timing of follow-up, activation protocol, measurement method, and outcome measured) and for the numerical data collection using pre-defined forms. Piloting of the forms was performed during the protocol stage until over 90% agreement was reached. When any data was missing in the trial, it was calculated from existing data or the corresponding author was contacted.

### Risk of bias in individual studies

The risk of bias within the individual included randomized studies was evaluated using the Cochrane risk of bias tool [19]. This assessment was performed by one author (MK) and independently checked by another author (SNP).

### Data synthesis

The primary outcome of this systematic review was the difference in the achieved amount of skeletal maxillary expansion between bone-borne or hybrid tooth-bone-borne RME and conventional tooth-borne RME. Secondary outcomes included dental positional/inclination changes, other skeletal changes, root resorption, structural/functional airway measurements, and patient-reported outcomes.

Data were summarized and considered suitable for pooling, if similar intervention and/or control groups were compared and if similar outcomes were reported. All existing trials were included in the analysis independently of reporting completeness, if possible; where data was missing, they were calculated from existing data or requested them from the authors. For studies reporting on data before and after treatment, but not on the treatment-induced changes, we calculated those with a moderate pre-post correlation of 0.75.

Mean differences (MDs) of treatment changes for continuous outcomes and relative risks (RRs) for binary outcomes and their corresponding 95% confidence intervals (CIs) were calculated. The standardized mean difference was also chosen post hoc to combine two similar measurements of nasal cavity width into a single meta-analysis (Additional file 1: Appendix 2). As the effects of RME were deemed to be highly variable according to patient age, sex, and individual variation of the maxillofacial sutures, a random effects model was chosen over a fixed effect one to calculate the average distribution of treatment effects that can be expected [21]. A REstricted Maximum Likelihood (REML) random effects variance estimator was used instead of the older DerSimonian-Laird one, following recent guidance [22]. Random effects 95% predictions were calculated for meta-analyses with at least three studies to aid in their interpretation by quantifying expected treatment effects in a future clinical setting [23].

The extent and impact of between-study heterogeneity were assessed by inspecting the forest plots and by calculating the tau-squared and the *I*-squared statistics, respectively. The 95% CIs (uncertainty intervals) around tau-squared and *I*-squared were calculated to judge our confidence about these metrics. We arbitrarily adopted the *I*-squared thresholds of > 75% to be considered as signs of considerable heterogeneity, but we also judged the evidence for this heterogeneity (through the uncertainty intervals) and the localization on the forest plot.

A two-tailed *P* value of 0.05 was considered significant for all hypothesis testing, except for a 0.10 used for the test of heterogeneity and reporting biases. All analyses were run in Stata SE 14.0 (StataCorp, College Station, TX) by one author (SNP), and the study’s dataset was openly provided [24].

### Risk of bias across studies and additional analyses

Subgroup analyses, meta-regressions, assessments of reporting biases, and sensitivity analyses were initially planned in the review’s protocol, but could ultimately not be conducted due to limited number of included trials (Additional file 1: Appendix 2).

The overall quality of clinical recommendations (confidence in effects estimates) for each of the main outcomes was rated by using the Grades of Recommendation, Assessment, Development, and Evaluation (GRADE) approach [25] using an improved summary of findings table format [26]. The optimal information size was estimated for each outcome independently to be able to identify a minimal clinical important effect with an average standard deviation (based on this review’s study sample), with type I and type II errors set at 5% and 20%, respectively. The minimal clinical important, large, and very large effects were conventionally defined as half, one, and two standard deviations for continuous outcomes [27] and as relative risks of 1.5, 2.5, or 5.0 for binary outcomes [28]. This assessment of the risk of bias for among trials was conducted independently by two authors (SNP and MK), and discrepancies were resolved by discussion with a third author (TE).

## Results

### Study selection

**Fig. 1:**
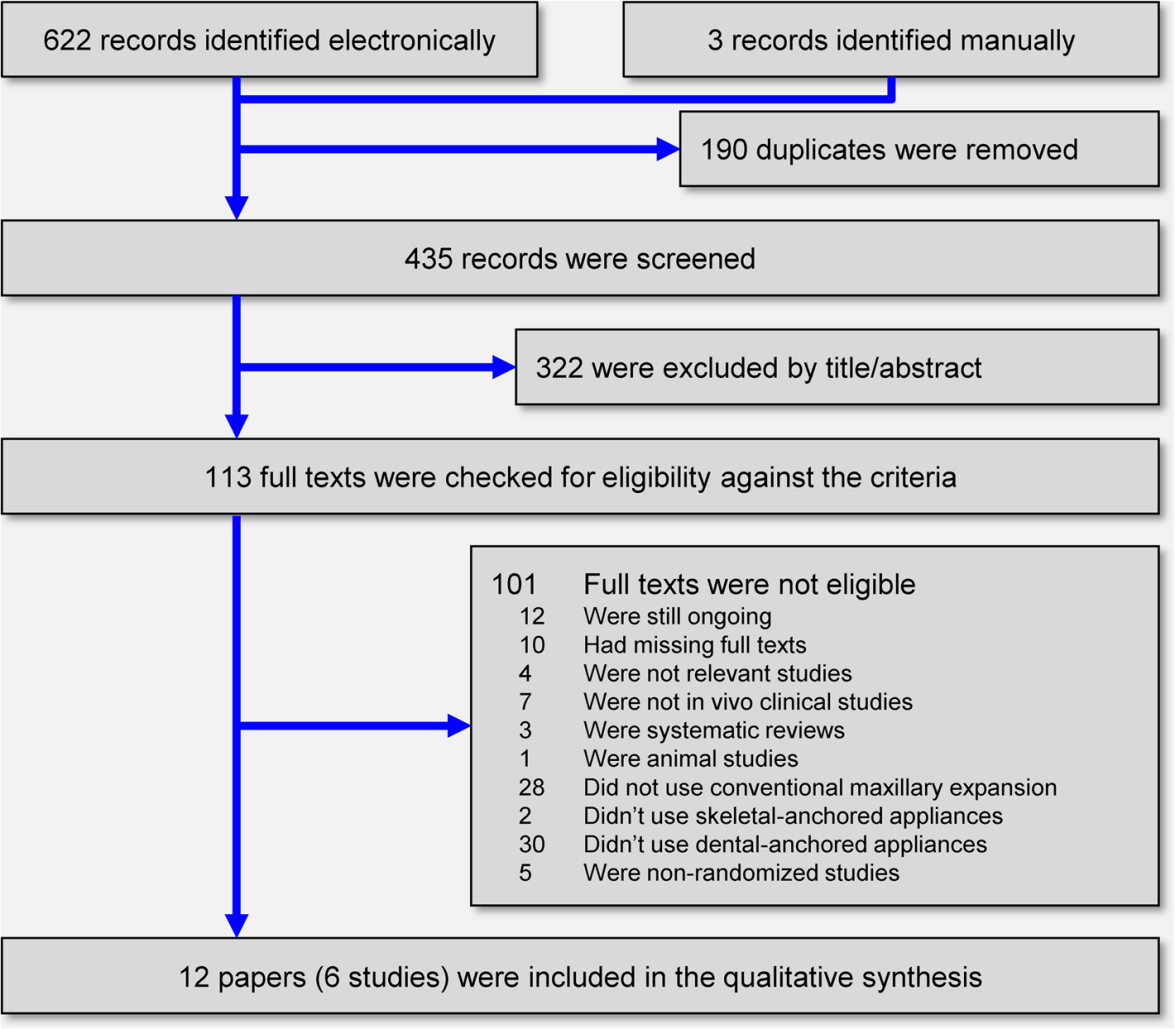
PRISMA flow diagram for identification and selection of eligible trials

The electronic literature yielded a total of 622 records, while 3 more were identified manually (Fig. 1). After removal of duplicates and screening of titles and abstracts, 113 full-text papers were scrutinized against the eligibility criteria. After applying the eligibility criteria, a total of 12 publications pertaining to six unique RCTs were finally included in this systematic review (Additional file 1: Appendix 3).

### Study characteristics

The six included RCTs were conducted in clinics, private practices, or university clinics in four different countries (Canada, Netherlands, Sweden, Turkey) and had been published as journal papers and/or dissertations in English between 2009 and 2018 (Table 1). As far as experimental groups are concerned, two trials included a pure bone-borne RME, three included a hybrid tooth-bone-borne RME, and one included both. The different designs of RME appliances used can be seen in Additional file 1: Appendix 4. As far as control groups are concerned, all six trials included a conventional tooth-borne RME, while one trial also included an untreated control group that was disregarded, as it fell outside the scope of this review. These six trials included a total of 264 patients randomized into experimental or control groups with an average group size of 19 patients. From these 264 patients, 112 (42.4%) were male and the average age across trials was 12.3 years. All trials used similar RME activation protocols, which included 2 turns of the expansion screw per day until (over-)correction of the maxillary deficit.

**Table 1:** Characteristics of included randomized trials pertaining to setting, patients, and intervention

Study	Design; Setting; Country$	Patients (M/F); age	Intervention; duration^#^	Activation protocol
Bazargani [33]	RCT; Clinic; SWE	EG1: 19 (11/8); 9.7 EG2: 21 (10/11); 10.2	EG1: TB RME; NR EG2: Hybr. RME; NR	2×/day until upper palatal molar cusps touch lower molar buccal cusps
Canan [31]	RCT; Uni; TUR	EG1: 16 (8/8); 12.6 EG2: 16 (7/9); 12.9 EG3: 15 (7/8); 13.4	EG1: TB RME; 13.3 days EG2: BB RME; 12.4 days EG3: Hybr. RME; 14.1 days	2×/day
Celenk-Koca [30]	RCT; Pract; NLD	EG1: 20 (8/12); 13.8 EG2: 20 (7/13); 13.8	EG1: TB RME; 19.7 days EG2: BB RME; 19.7 days	2×/day until upper palatal molar cusps touch lower molar buccal cusps
Feldmann [37]	RCT; Clinic; SWE	EG1: 25 (12/13); 9.7 EG2: 25 (12/13); 10.0	EG1: TB RME; NR EG2: Hybr. RME; NR	2×/day until upper palatal molar cusps touch lower molar buccal cusps
Gunyuz Toklu [18]	RCT; Uni; TUR	EG1: 13 (5/8); 14.3 EG2: 12 (6/6); 13.8	EG1: TB RME; 19.2 days EG2: Hybr. RME; 20.2 days	2×/day until upper palatal molar cusps touch lower molar buccal cusps
Lagravère 2009 [45]_collated_^†^	RCT; Uni; CAN	EG1: 20 (5/15); 14.1 EG2: 21 (8/13); 14.2 CG: 21 (6/15); 12.9	EG1: TB RME; NR EG2: BB RME; NR CG: observation	2×/day for DME (or 1×/2 days for the SME) until overcorrection

*BB* bone-borne, *CG* control group without expansion, *EG* experimental group with expansion, *F* female, *Hybr.* hybrid (tooth-bone-borne), *RCT* randomized clinical trial, *HME* hybrid (skeletally/dentally) anchored maxillary expansion, *M* male, *NR* not reported, *Pract* practice, *RME* rapid maxillary expansion, *SME* skeletally anchored maxillary expansion, *TB* tooth-borne, *Uni* university

^#^Duration of active transverse expansion in weeks

^$^Countries are given with their ISO-3 code

^†^Including the publications Lagravère 2010 [46], Lagravère 2013 [47], Kabalan 2015 [48], Stepanko 2016 [49] and the dissertations Lagravère 2009 [45], Forst 2015 [36]

As far as outcome measurement is concerned (Table 2), one trial assessed patient-reported outcomes during the first expansion days, three trials assessed outcomes directly post-expansion, and four trials assessed outcomes after an additional retention/observation period. A wide variety of outcomes were measured by cone beam computerized tomography (CBCT) (four trials), rhinomanometry (two trials), plaster cast models (one trial), and questionnaires (one trial).

**Table 2:** Characteristics of included randomized trials pertaining to follow-up and outcome

Study	Follow-up	Method/outcome
Bazargani [33]	Post-exp	Rhinomanometry ▪ Nasal airflow ▪ Nasal resistance Plaster casts ▪ Dental arch width (IMW)
Canan [31]	• Post-exp • 6.0 months post-exp	CBCT ▪ 3D tooth movements ▪ Dental arch width (ICW, IP1W, IMW) ▪ Dental tipping (P1, M) Clinical ▪ Technical complications
Celenk-Koca [30]	• 6.0 months post-exp	CBCT ▪ Dental arch width (IP1W, IMW) ▪ Dental tipping (P1, M) ▪ Root resorption ▪ Skeletal maxillary width ▪ Skeletal sutural opening amount & pattern
Feldmann [37]	1st/4th exp day	Questionnaire ▪ Pain ▪ Discomfort ▪ Jaw function ▪ Analgesic consumption
Gunyuz Toklu [18]	3.0 mos post-exp	CBCT ▪ Facial width ▪ Skeletal maxillary width ▪ Buccal/palatal bone thickness (C, P1, P2, M) ▪ Alveolar (P1, M) / dental (C, P1, P2, M) tipping ▪ Dental arch width (ICW, IP1W, IP2W, IMW)
Lagravère 2009 [45]_collated_^†^	• Post-exp • 6.0 months post-exp • 12.0 months post-exp	CBCT ▪ Nasal width ▪ Skeletal maxillary width ▪ Skeletal mandibular width ▪ Dental arch width (IP1W, IMW) ▪ Sagittal/vertical tooth movements (I, M) ▪ Sagittal/vertical skeletal mandibular position Questionnaire ▪ Pain Acoustic rhinometry ▪ Nasal airway volume

*CBCT* cone beam computed tomography, *Exp* expansion, *I* incisor, *IMW* intermolar width, *IP1W* inter-(first)-premolar width, *M* molar, *P1* first premolar, *P2* second premolar

^†^Including the publications Lagravère 2010 [46], Lagravère 2013 [47], Kabalan 2015 [48], Stepanko 2016 [49] and the dissertations Lagravère 2009 [45], Forst 2015 [36]

### Risk of bias within studies

**Fig. 2:**
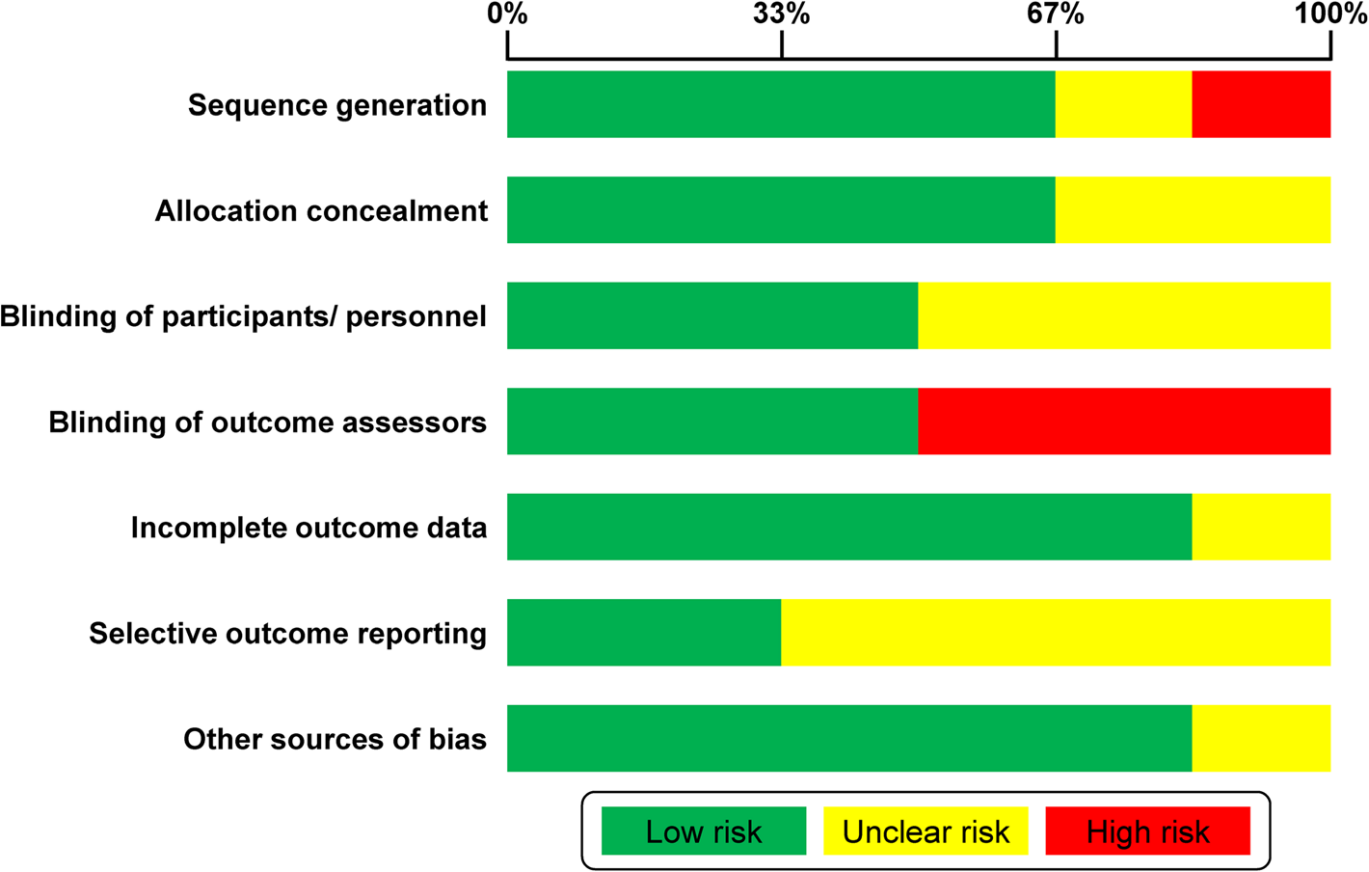
Risk of bias summary for included randomized trials with the Cochrane tool

The risk of bias of included trials ranged between low (one trial), unclear/low (two trials), and high (three trials). The most frequent reason for assigning a high risk for bias was the lack of blinding for the outcome measurement (three trials), followed by a potentially inadequate generation of the randomization sequence (one trial) (Fig. 2; Additional file 1: Appendix 5).

### Results of individual studies and synthesis of results

The results of every extracted outcome from each included randomized trial are given in Additional file 1: Appendix 6a for the comparison of bone-borne versus tooth-borne expanders and in Additional file 1: Appendix 6b for the comparison of hybrid (tooth-bone-borne) versus tooth-borne expanders. Only statistically significant and clinically relevant differences are given here, which pertain to results of single trials, unless meta-analyses are available, where they are reported as such and given in Table 3.

**Table 3:** Results of random effects meta-analyses performed from randomized trials comparing tooth-borne rapid maxillary expansion with either bone-borne or hybrid (tooth-bone-borne) rapid maxillary expansion

Experimental	Timing	Outcome	Trials	MD	95% CI	*P*	*I*^2^ (95% CI)	*τ*^2^ (95% CI)	95% PrI
Bone-borne	Pst-Exp	Intermolar width (crown)	2	− 0.09	− 0.34, 0.16	0.46	0% (0%, 98%)	0 (0, 8.14)	NC
Bone-borne	Pst-Exp	Inter-1st-premolar width (crown)	2	− 0.71	− 2.70, 1.27	0.48	91% (41%, 100%)	1.88 (0.12, 258.72)	NC
Bone-borne	Pst-Exp	Inclination 1st molar (left)	2	− 2.93	− 7.87, 2.01	0.25	83% (0%, NC)	10.59 (0, NC)	NC
Bone-borne	Pst-Exp	Inclination 1st molar (right)	2	− 1.47	− 3.90, 0.95	0.23	0% (0%, 98%)	0 (0, 189.07)	NC
Bone-borne	Pst-Exp	Inclination 1st premolar (left)	2	− 2.49	− 5.19, 0.22	0.07	60% (0%, 100%)	2.31 (0, 483.98)	NC
Bone-borne	Pst-Exp	Inclination 1st premolar (right)	2	− 4.05	− 5.97, − 2.13	< 0.001	0% (0%, 98%)	0 (0, 93.81)	NC
Bone-borne	Reten	Intermolar width (crown)	3	0.15	− 0.27, 0.56	0.49	0% (0%, 88%)	0 (0, 1.23)	− 2.54, 2.83
Bone-borne	Reten	Inter-1st-premolar width (crown)	3	− 0.66	− 1.90, 0.58	0.30	77% (0%, 99%)	0.88 (0, 22.64)	− 15.06, 13.74
Bone-borne	Reten	Inclination 1st molar (left)	2	− 1.89	− 9.48, 5.70	0.63	87% (10%, NC)	26.04 (0.45, NC)	NC
Bone-borne	Reten	Inclination 1st molar (right)	2	− 0.20	− 3.91, 3.51	0.92	59% (0%, 100%)	4.33 (0, 913.80)	NC
Bone-borne	Reten	Inclination 1st premolar (left)	2	− 2.38	− 9.53, 4.76	0.51	90% (31%, NC)	23.94 (1.24, NC)	NC
Bone-borne	Reten	Inclination 1st premolar (right)	2	− 0.77	− 3.02, 1.48	0.50	0% (0%, 98%)	0 (0, 154.35)	NC
Bone-borne	Reten	Nasal cavity width	2	*0.41	*− 0.03, 0.84	0.07	0% (0%, 99%)	0 (0, 7.60)	NC
Hybrid	Reten	Intercanine width (crown)	2	− 0.22	− 0.98, 0.55	0.58	36% (0%, 100%)	0.16 (0, 55.23)	NC
Hybrid	Reten	Intermolar width (crown)	2	0.18	− 0.40, 0.76	0.55	0% (0%, 98%)	0 (0, 28.03)	NC
Hybrid	Reten	Inter-1st-premolar width (crown)	2	− 1.96	− 6.18, 2.27	0.36	89% (27%, NC)	8.36 (0.38, NC)	NC
Hybrid	Reten	Inclination 1st molar (left)	2	− 1.29	− 3.61, 1.03	0.28	0% (0%, 99%)	0 (0, 341.30)	NC
Hybrid	Reten	Inclination 1st molar (right)	2	− 1.12	− 6.96, 4.72	0.71	66% (0%, NC)	11.75 (0, NC)	NC
Hybrid	Reten	Inclination 1st premolar (left)	2	− 3.97	− 7.08, − 0.86	0.01	49% (0%, 100%)	2.70 (0, 685.54)	NC
Hybrid	Reten	Inclination 1st premolar (right)	2	− 0.79	− 3.18, 1.60	0.52	0% (0%, 98%)	0 (0, 181.80)	NC

*CI* confidence interval, *MD* mean difference, *NC* non-calculable, *PrI* predictive interval, *Pst-Exp* post expansion, *Reten* post retention period (at least 3 months)

*Pertains to standardized mean difference, as two similar outcomes were pooled together: nasal cavity width at orbita and nasal cavity width at the 1st premolar

#### Bone-borne compared with tooth-borne rapid maxillary expansion

As far as differences directly post-expansion are concerned (Additional file 1: Appendix 6a), only some dental positional/inclinational significant differences were reported from a single trial. As such, bone-borne RME was associated with less dental expansion at the canine (MD − 0.7 mm; 95% CI − 1.0 to − 0.4 mm), less buccal tipping at the first premolar (MD − 4.3°; 95% CI − 6.9 to − 1.6°), and less buccal tipping at the first molar (MD − 5.4°; 95% CI − 8.0 to − 2.7°) compared to tooth-borne RME. Additionally, a meta-analysis of two trials indicated that bone-borne RME was associated with less buccal tipping of the first premolar (MD − 4.1°; 95% CI − 6.0 to − 2.1°).

As far as data after a retention/observation period post-expansion are concerned (Additional file 1: Appendix 6a), several skeletal maxillary, dental positional/inclinational, and nasal cavity clinically relevant differences were identified. One trial indicated that bone-borne RME was associated with (i) greater skeletal expansion at the incisal foramen (MD 1.8 mm; 95% CI 1.3 to 2.3 mm), (ii) greater suture opening at the first premolar (MD 2.3 mm; 95% CI 1.7 to 2.9 mm), and (iii) greater suture opening at the first molar (MD 2.0 mm; 95% CI 1.4 to 2.6 mm) than tooth-borne RME. Three different single trials provided evidence that bone-borne RME was associated with (i) less intercanine width expansion (MD − 0.5 mm; 95% CI − 1.0 to − 0.1 mm), (ii) less inter-first-premolar width expansion (MD − 1.8 mm; 95% CI − 2.7 to − 0.9 mm), (iii) less buccal inclination of the first premolar (MD − 5.1°; 95% CI − 6.8 to − 3.4°), (iv) less buccal inclination of the first molar (MD − 5.2°; 95% CI − 7.0 to − 3.5°), and (v) greater buccal bone thickness at the 1st premolar (MD 0.3 mm; 95% CI 0.1 to 0.4 mm) than tooth-borne RME. Additionally, bone-borne RME was associated with greater expansion of the nasal cavity width at the first molar (MD 1.7 mm; 95% CI 0.8 to 2.6 mm) than tooth-borne RME. Finally, no clinically relevant differences regarding skeletal vertical dimension, mandibular dimensions, or root resorption were observed.

#### Hybrid (tooth-bone-borne) compared with tooth-borne rapid maxillary expansion

As far as differences directly post-expansion are concerned (Additional file 1: Appendix 6b), only one trial indicated that hybrid RME was associated with less intercanine width expansion (MD − 0.7 mm; 95% CI − 0.9 to − 0.4 mm) compared to tooth-borne RME.

As far as differences after a retention/observation period are concerned, one trial indicated that hybrid RME was associated with (i) less inter-first-premolar width expansion (MD − 4.3 mm; 95% CI − 7.0 to − 1.6 mm), (ii) less inter-second-premolar width expansion (MD − 3.3 mm; 95% CI − 6.2 to − 0.5 mm), (iii) greater buccal bone thickness at the first premolar (MD 0.8 mm; 95% CI 0.3 to 1.3 mm), and (iv) lower palatal bone thickness at the first premolar (MD − 1.6 mm; 95% CI − 2.2 to − 1.0 mm) than tooth-borne RME. Finally, one meta-analysis of two trials indicated that hybrid RME was associated with less buccal tipping of the first premolar (MD − 4.0 mm; 95% CI − 7.1 to − 0.9 mm) than tooth-borne RME.

### Risk of bias across studies and additional analyses

No formal assessment of risk of bias across studies or any subgroup/sensitivity analyses could be performed due to the limited number of included trials in the meta-analyses, which would be rendered instable by trial omissions.

The quality of evidence for the comparison of bone-borne versus tooth-borne RME varied between very low and moderate (Table 4). Moderate quality of evidence supported the greater sutural opening at the first premolar and the first molar, low quality of evidence backed the change in nasal cavity width or root resorption, while very low quality of evidence supported dental tipping changes during RME. The main reasons for downgrading were (i) the imprecision due to inadequate sample sizes of all trials, (ii) bias due to lack of outcome measurement blinding and inadequate randomization sequence generation, and (iii) inconsistency due to high heterogeneity.

**Table 4:** Summary of findings table according to the GRADE approach for the comparison of bone-borne versus tooth-borne rapid maxillary expansion

	Anticipated absolute effects^a^ (95% CI)				
Outcome Trials (patients)	Tooth-borne RME^b^	Bone-borne RME	Difference	Quality of the evidence (GRADE)^c^	What happens
Suture opening at 1st premolar Post-retention 40 patients (1 trial)	1.3 mm	–	2.3 mm more (1.7 to 2.9 more)	⊕⊕⊕⃝○ moderate^d^ due to imprecision	Probably greater sutural opening with bone-borne RME
Suture opening at 1st molar Post-retention 40 patients (1 trial)	1.1 mm	–	2.0 mm more (1.4 to 2.6 more)	⊕⊕⊕○⃝ moderate^d^ due to imprecision	Probably greater sutural opening with bone-borne RME
Buccal tipping of 1st premolar Post-retention 73 patients (2 trials)	3.9°	–	2.4° less (9.5 less to 4.8 more)	⊕⃝⃝⃝○○○ very low^d, e, f^ due to bias, inconsistency, imprecision	Little to no difference in premolar buccal tipping
Buccal tipping of 1st molar Post-retention 73 patients (2 trials)	5.7°	–	1.9° less (9.5 less to 5.7 more)	⊕⃝⃝⃝○○○ very low^d, e, f^ due to bias, inconsistency, imprecision	Little to no difference in molar buccal tipping
Nasal cavity width at 1st premolar/orbita^$^ Post-retention 81 patients (2 trials)	1.8 mm^$^	–	0.7 mm more(0.1 less to 1.4 more)	⊕⊕⃝⃝○○ low^d, e^ due to bias, imprecision	Little to no difference in nasal cavity width
Root resorption volume at 1st molar Post-retention 41 patients (1 trial)	49.3 mm^3^	–	17.8 mm^3^ less (46.0 to 10.4 more)	⊕⊕⃝⃝○○ low^d, e^ due to bias, imprecision	Little to no difference in root resorption volume

Bone-borne versus tooth-borne rapid maxillary expansion

Population and intervention: adolescent or adult patients with skeletal maxillary deficit

Settings: university clinics, private practices, and clinics (Canada, Netherlands, Sweden, Turkey)

*CI* confidence interval, *GRADE* Grading of Recommendations Assessment, Development and Evaluation

^a^The basis for the risk in the control group (e.g., the median control group risk across studies) is provided in footnotes. The risk in the intervention group (and its 95% confidence interval) is based on the assumed risk in the comparison group and the relative effect of the intervention (and its 95% CI)

^b^Response in the control group is based on average response of included trials

^c^Starts from “high,” due to the inclusion of randomized studies

^d^Downgraded by one point due to imprecision, as the optimal information size was judged not to be met

^e^Downgraded by one point for risk of bias (lack of blind outcome assessment)

^f^Downgraded one for inconsistency (*I*^2^ > 75%)

^$^Standardized mean difference was used for the meta-analysis and was back-translated to natural units based on the data from the Celenk-Koca 2018 [30] trial

The quality of evidence for the comparison of hybrid tooth-bone-borne versus tooth-borne RME varied between low and moderate (Table 5). Apart from the change in the external maxillary skeletal width that was supported by low quality of evidence, all other comparisons were backed by moderate quality of evidence. The main reasons for downgrading were (i) the imprecision due to inadequate sample sizes of all trials and (ii) bias due to lack of outcome measurement blinding and inadequate randomization sequence generation.

**Table 5:** Summary of findings table according to the GRADE approach for the comparison of hybrid (tooth-bone-borne) versus tooth-borne rapid maxillary expansion

Outcome Trials (patients)	Anticipated absolute effects^a^ (95% CI)					
	Relative effect (95% CI)	Tooth-borne RME^b^	Hybrid (tooth-bone-borne) RME	Difference	Quality of the evidence (GRADE)^c^	What happens
External maxillary width at 1st molar Post-retention 25 patients (1 trial)	–	2.0 mm	–	0.6 mm more (1.4 less to 2.7 more)	⊕⃝⃝⃝○○○ low^d, e^ due to bias, imprecision	Little to no difference in external maxillary width at 1st molars
Buccal tipping of 1st premolar Post-retention 56 patients (2 trials)	–	3.7°	–	4.0° less (0.9 to 7.1 less)	⊕⊕⃝⃝○○ moderate^e, f^ due to bias, imprecision	Probably less premolar tipping with hybrid RME
Buccal tipping of 1st molar Post-retention 56 patients (2 trials)	–	4.3°	–	1.3° less (3.6 less to 1.0 more)	⊕⊕⃝⃝○○ moderate^e, f^ due to bias, imprecision	Little to no difference in molar buccal tipping
Nasal resistance Post-expansion 30 patients (1 trial)	–	0.9 Pa s/cm^3^	–	0.2 Pa s/cm^3^ less(0 to 0.4 less)	⊕⊕⊕⃝○ moderate^e^ due to imprecision	Probably lower nasal resistance with hybrid RME
Analgesic use on 1st expansion day Post-retention 50 patients (1 trial)	RR 0.8 (0.3 to 1.8)	36.0%	28.1% (12.2 to 63.4%)	7.9% less (13.8% less to 27.4% more)	moderate^e^ ⊕⊕⊕⃝⃝○ due to imprecision	Little to no difference in analgesic use

Bone-borne versus tooth-borne rapid maxillary expansion

Population and intervention: adolescent or adult patients with skeletal maxillary deficit

Settings: university clinics, private practices, and clinics (Canada, Netherlands, Sweden, Turkey)

*CI* confidence interval, *GRADE* Grading of Recommendations Assessment, Development and Evaluation

^a^The basis for the risk in the control group (e.g., the median control group risk across studies) is provided in footnotes. The risk in the intervention group (and its 95% confidence interval) is based on the assumed risk in the comparison group and the relative effect of the intervention (and its 95% CI)

^b^Response in the control group is based on average response of included trials

^c^Starts from “high,” due to the inclusion of randomized studies

^d^Downgraded by two points due to risk of bias (potentially inadequate randomization and lack of blind outcome assessment)

^e^Downgraded by one point due to imprecision, as the optimal information size was judged not to be met

^f^Downgraded by one point for risk of bias (lack of blind outcome assessment)

## Discussion

### Summary of evidence

The present systematic review summarizes and critically appraises evidence from randomized clinical trials on the potential benefits of partly or completely skeletally anchored RME compared to conventional RME and is to our knowledge the first review to do so. A total of 12 publications pertaining to six unique trials including a total of 264 patients in need of RME were finally included in the analyses. The quality of recommendations that can be drawn from existing evidence according to the GRADE approach varies between very low to moderate, as all are based on few trials with mostly inadequate sample sizes and some trials have methodological limitations. This means that our confidence in these recommendations is hampered and future trials might change these provisional recommendations.

The idea behind using skeletal anchorage for RME is that greater skeletal expansion of the maxilla could theoretically be obtained. This systematic review found that bone-borne RME was associated with greater opening of the maxillary suture at the incisal foramen (1.8 mm more), first premolars (2.3 mm more), and the first molars (2.0 mm more) compared to conventional RME (Additional file 1: Appendix 6a). This might be explained by a direct force application to the maxilla, which leads to separation of the median suture and displacement of the two maxillary halves [29]. Sutural opening was not assessed for hybrid RME in any of the identified trials. Interestingly, no significant increase in the external buccal maxillary width at the first molars of either bone-borne (*P* = 0.22; Additional file 1: Appendix 6a) or hybrid RME (*P* = 0.54; Additional file 1: Appendix 6b) was seen compared to conventional RME. This might be attributed to either bone remodeling or alveolar bending [18].

At the same time, skeletally anchored RME is proposed over tooth-borne RME by many as a means of reducing the adverse effect of buccal tipping of the anchorage teeth. Evidence from the present review was inconclusive on whether bone-borne or hybrid RME could prevent buccal tooth tipping to a clinically meaningful degree. Even though the effects for the first premolar or first molar for both types of skeletally anchored RMEs were < 0 (indicating less tipping than conventional RME), this was mostly not statistically significant (Table 3). However, caution is warranted in the interpretation of these findings, since this might be attributed to the limited number of trials with small sample sizes and heterogeneous results or to the fact that studies measured this outcome separately for right and left teeth. An indirect way to measure maxillary expansion in conjunction with tooth tipping might be to look at the buccal bone thickness at the first premolars and the first molars. Here, evidence from one trial [30] indicated that bone-borne RME was associated with significantly greater buccal bone thickness at the first premolar and first molar (0.14 mm and 0.25 mm, respectively; Additional file 1: Appendix 6a) compared to conventional tooth-borne RME. On the other side, data on buccal bone thickness after hybrid RME were more inconclusive, indicating greater bone thickness at the first premolar (0.63 mm more), but not the first molar (Additional file 1: Appendix 6b) [18]. This might be attributed to the fact that the hybrid RME appliance was anchored on the first molars, but not the first premolars. When analyzing dental tipping after RME, it is important to bear in mind the pyramid- or triangle-shaped opening of the suture due to the two centers of rotation, which leads to bending of the alveolar bone and subsequent tipping of the teeth [18, 31]. Therefore, there are some indications that tipping of the anchorage teeth might be influenced by anchorage type, although further evidence is needed to consolidate these.

Another outcome often measured in trials comparing bone-borne or hybrid RME to conventional RME is the dental arch width—usually at the first premolars or the first molars. Existing data indicated no significant difference in this dental arch width for either skeletally anchored RME compared to conventional RME (*P* > 0.05; Table 3). However, this is to be expected, since the most widely used criterion to terminate RME was clinically determined when the upper palatal molar cusps touched the lower molar buccal cusps.

Several studies have reported increased width of the nasal cavity post expansion, which seems to be associated with the opening of the midpalatal suture [17, 32]. Evidence from a trial included in the present review indicated that bone-borne RME was associated with increased nasal cavity width at the first molar (by 1.7 mm; Additional file 1: Appendix 6a) compared to conventional RME [30]. Additionally, data from another trial indicated that hybrid RME was associated with increased nasal airflow (by 57.7 cm^3^/s; Additional file 1: Appendix 6b) and reduced nasal airway resistance (by − 0.2 Pa s/cm^3^; Additional file 1: Appendix 6b) compared to conventional RME [33]. Although it has been shown that RME in general might potentially increase the volume of the upper airways [34], the effect to which this contributes in improved breathing or quality of life in unclear. It must be noted here that based on current evidence no recommendations can be made for the use of any kind of RME for the treatment of breathing disorders like obstructive sleep apnea [35].

Another side effect of RME that could potentially be alleviated using skeletal anchorage is the iatrogenic root resorption of the premolars and molars used as anchorage for the RME appliance [16]. Two trials included in the present review using bone-borne RME found no considerable differences in either linear or volumetric root resorption compared to conventional RME [30, 36].

Finally, as patient reported outcomes are concerned, only one trial existed that compared the short-term effect on the pain and discomfort during the first week of RME with a hybrid and a conventional appliance [37]. The results indicated that no significant differences exist in the pain or discomfort and analgesic consumption, apart from pain from molars/incisors and tensions from the jaw on day 4, where the hybrid RME group reported less disturbances than the conventional RME group.

### Strengths and limitations

The strengths of this systematic review consist of the registration of its a priori protocol in PROSPERO [38, 39], its exhaustive literature search, its improved analytical methods [22], the use of the GRADE approach [25] to assess the quality of the meta-evidence, and the transparent provision of the study’s data [24, 40].

However, certain limitations also exist. First and foremost, although only randomized trials were included that are generally less prone to bias than non-randomized trials [41], many of them had methodological limitations that might lead to bias [42]. Furthermore, the identified studies were predominantly small and this might introduce small-study effects [43]. Finally, the limited number of included studies and their suboptimal reporting did not enable assessments of heterogeneity, as well as the conduct of several analyses for subgroups (including among others different implant placement regions and different RME appliances), small-study effects, and reporting biases that were planned to assess the robustness of the analyses [44].

## Conclusions

Existing evidence from randomized trials on RME for transverse maxillary deficit indicates that bone-borne RME might be associated with greater skeletal maxillary expansion post-retention compared to tooth-borne RME, while no significant differences could be identified for a buccal tooth tipping, nasal cavity width, and root resorption. Hybrid tooth-bone-borne RME was associated with less patient discomfort during the first days of activation, less buccal tipping of the first premolar and lower nasal airway resistance post-retention compared to tooth-borne RME, while no significant differences could be found regarding skeletal maxillary width, molar inclination, and analgesic use. Overall, there exist some indications of potential benefits from partially or completely skeletally anchored RME, but only a few trials with very limited sample sizes and some risk of bias exist, which hampers our confidence in drawing clinical recommendations. Future well-designed randomized trials with a priori sample size calculation and blinded assessment of skeletal, dental, and breathing-related outcomes are needed.

## Additional file

Additional file 1: Appendix 1. Literature searched conducted to identify eligible studies (last search date September 28, 2018). Appendix 2. Additional information about this review, including deviations from protocol. Appendix 3. List of studies identified from the literature and their inclusion/exclusion status, with reasons. Appendix 4. Details of the expansion appliances used in the included trials. Appendix 5. Risk of bias assessment of included randomized trials. Appendix 6a. List of included trials comparing bone-borne with tooth-borne rapid maxillary expansion. Appendix 6b. List of included trials comparing hybrid (tooth-bone-borne) with tooth-borne rapid maxillary expansion. (PDF 694 kb)

## Abbreviations

CBCT: Cone beam computerized tomography
CI: Confidence interval
GRADE: Grading of Recommendations Assessment, Development and Evaluation
MD: Mean difference
PICOS: Participants-Interventions-Comparisons-Outcome-Study design
RCT: Randomized clinical trial
REML: REstricted Maximum Likelihood
RME: Rapid maxillary expansion
RR: Relative risk
