# Supplementary material for: Are there benefits from using bone-borne maxillary expansion instead of tooth-borne maxillary expansion? A systematic review with meta-analysis
Source: Prog Orthod. 2019 Feb 25;20:9. doi: 10.1186/s40510-019-0261-5 (PMC6387979; doi:10.1186/s40510-019-0261-5)
Supplement: Supplementary file 1 — Appendix 1. Literature searched conducted to identify eligible studies (last search date September 28, 2018). Appendix 2. Additional information about this review, including deviations from protocol. Appendix 3. List of studies identified from the literature and their inclusion/exclusion status, with reasons. Appendix 4. Details of the expansion appliances used in the included trials. Appendix 5. Risk of bias assessment of included randomized trials. Appendix 6a. List of included trials comparing bone-borne with tooth-borne rapid maxillary expansion. Appendix 6b. List of included trials comparing hybrid (tooth-bone-borne) with tooth-borne rapid maxillary expansion. (PDF 694 kb) [file 40510_2019_261_MOESM1_ESM.pdf]

Appendix 1. Literature searched conducted to identify eligible studies (last search date September 28th, 2018).

Search for clinical studies				Search for randomized studies		
**Database**	**Search**	**Limits**	**Hits**	**Database**	**Search**	**Hits**
MEDLINE (via PubMed)	("maxillary expansion" OR ((expand* OR expans*) AND maxill*)) AND ("bone-anchorage" OR "bone-anchored" OR "bone-borne" OR "implant-anchorage" OR "implant-anchored" OR "implant-borne" OR "mini-implant" OR "minipin" OR "mini-pin" OR "miniscrew" OR "mini-screw" OR "mini-implant" OR "palatal implant" OR "skeletal anchorage" OR "skeletally-anchored" OR "transpalatal distraction")	Humans	177	MEDLINE (via PubMed)	("maxillary expansion" OR ((expand* OR expans*) AND maxill*)) AND ("bone-anchorage" OR "bone-anchored" OR "bone-borne" OR "implant-anchorage" OR "implant-anchored" OR "implant-borne" OR "mini-implant" OR "minipin" OR "mini-pin" OR "miniscrew" OR "mini-screw" OR "mini-implant" OR "palatal implant" OR "skeletal anchorage" OR "skeletally-anchored" OR "transpalatal distraction") AND (randomized controlled trial[pt] OR controlled clinical trial[pt] OR randomized[tiab] OR placebo[tiab] OR clinical trials as topic[mesh:noexp] OR randomly[tiab] OR trial[ti] NOT (animals[mh] NOT humans [mh]))	26
Embase	Same as MEDLINE	Human	30	Embase	("maxillary expansion" OR ((expand* OR expans*) AND maxill*)) AND ("bone-anchorage" OR "bone-anchored" OR "bone-borne" OR "implant-anchorage" OR "implant-anchored" OR "implant-borne" OR "mini-implant" OR "minipin" OR "mini-pin" OR "miniscrew" OR "mini-screw" OR "mini-implant" OR "palatal implant" OR "skeletal anchorage" OR "skeletally-anchored" OR "transpalatal distraction") AND ('crossover procedure':de OR 'double-blind procedure':de OR 'randomized controlled trial':de OR 'single-blind procedure':de OR random*:de,ab,ti OR factorial*:de,ab,ti OR crossover*:de,ab,ti OR ((cross NEXT/1 over*):de,ab,ti) OR placebo*:de,ab,ti OR ((doubl* NEAR/1 blind*):de,ab,ti) OR ((singl* NEAR/1 blind*):de,ab,ti) OR assign*:de,ab,ti OR allocat*:de,ab,ti OR volunteer*:de,ab,ti) AND [embase]/lim	8
CDSR	Same as MEDLINE		0	CDSR	("maxillary expansion" OR ((expand* OR expans*) AND maxill*)) AND ("bone-anchorage" OR "bone-anchored" OR "bone-borne" OR "implant-anchorage" OR "implant-anchored" OR "implant-borne" OR "mini-implant" OR "minipin" OR "mini-pin" OR "miniscrew" OR "mini-screw" OR "mini-implant" OR "palatal implant" OR "skeletal anchorage" OR "skeletally-anchored" OR "transpalatal distraction")	0
DARE	Same as MEDLINE		0	DARE	Same as CDSR	0
CENTRAL	Same as MEDLINE		30	CENTRAL	Same as CDSR	31
Scopus	(TITLE-ABS-KEY (("maxillary expansion" OR ((expand* OR expans*) AND maxill*))) AND TITLE-ABS-KEY (("bone-anchorage" OR "bone-anchored" OR "bone-borne" OR "implant-anchorage" OR "implant-anchored" OR "implant-borne" OR "mini-implant" OR "minipin" OR "mini-pin" OR "miniscrew" OR "mini-screw" OR "mini-implant" OR "palatal implant" OR "skeletal anchorage" OR "skeletally-anchored" OR "transpalatal distraction")))	Dentistry Human/humans	131	Scopus	#1: (INDEXTERMS ("clinical trials" OR "clinical trials as a topic" OR "randomized controlled trial" OR "Randomized Controlled Trials as Topic" OR "controlled clinical trial" OR "Controlled Clinical Trials" OR "random allocation" OR "Double-Blind Method" OR "Single-Blind Method" OR "Cross-Over Studies" OR "Placebos" OR "multicenter study" OR "double blind procedure" OR "single blind procedure" OR "crossover procedure" OR "clinical trial" OR "controlled study" OR "randomization" OR "placebo")) OR (TITLE-ABS-KEY (("clinical trials" OR "clinical trials as a topic" OR "randomized controlled trial" OR "Randomized Controlled Trials as Topic" OR "controlled clinical trial" OR "Controlled Clinical Trials as Topic" OR "random allocation" OR "randomly allocated" OR "allocated randomly" OR "Double-Blind Method" OR "Single-Blind Method" OR "Cross-Over Studies" OR "Placebos" OR "cross-over trial" OR "single blind" OR "double blind" OR "factorial design" OR "factorial trial"))) OR (TITLE-ABS (clinical trial* OR trial* OR rct* OR random* OR blind*))	
					#2: ("maxillary expansion" OR ((expand* OR expans*) AND maxill*)) AND ("bone-anchorage" OR "bone-anchored" OR "bone-borne" OR "implant-anchorage" OR "implant-anchored" OR "implant-borne" OR "mini-implant" OR "minipin" OR "mini-pin" OR "miniscrew" OR "mini-screw" OR "mini-implant" OR "palatal implant" OR "skeletal anchorage" OR "skeletally-anchored" OR "transpalatal distraction")	
					#1 AND #2	46
Web of Knowledge	Same as MEDLINE	DENTISTRY ORAL SURGERY MEDICINE	205	Web of Knowledge	#1: TS= clinical trial* OR TS=research design OR TS=comparative stud* OR TS=evaluation stud* OR TS=controlled trial* OR TS=follow-up stud* OR TS=prospective stud* OR TS=random* OR TS=placebo* OR TS=(single blind*) OR TS=(double blind*)	
					#2: ("maxillary expansion" OR ((expand* OR expans*) AND maxill*)) AND ("bone-anchorage" OR "bone-anchored" OR "bone-borne" OR "implant-anchorage" OR "implant-anchored" OR "implant-borne" OR "mini-implant" OR "minipin" OR "mini-pin" OR "miniscrew" OR "mini-screw" OR "mini-implant" OR "palatal implant" OR "skeletal anchorage" OR "skeletally-anchored" OR "transpalatal distraction")	79
Virtual Health Library	Same as MEDLINE		19	Virtual Health Library	("maxillary expansion" OR ((expand* OR expans*) AND maxill*)) AND ("bone-anchorage" OR "bone-anchored" OR "bone-borne" OR "implant-anchorage" OR "implant-anchored" OR "implant-borne" OR "mini-implant" OR "minipin" OR "mini-pin" OR "miniscrew" OR "mini-screw" OR "mini-implant" OR "palatal implant" OR "skeletal anchorage" OR "skeletally-anchored" OR "transpalatal distraction") AND random*	0
ClinicalTrials.gov	("maxillary expansion" OR ((expand* OR expans*) AND maxill*))		17	ClinicalTrials.gov	("maxillary expansion" OR ((expand* OR expans*) AND maxill*))	17
**SUM (with overlap)**			432	**SUM (with overlap)**		**207**

CDSR, Cochrane Database of Systematic Reviews; CENTRAL, Cochrane Central Register of Controlled Trials; DARE, Database of Abstracts of Reviews of Effects.

Appendix 2. Additional information about this review, including deviations from protocol.

Deviations from protocol

- We decided post hoc to use the standardized mean difference to combine to similar measurements of the nasal cavity width (at the premolars and at the orbita). This is reported as a post hoc decision.
- The number needed to treat was planned to be calculated to clinically translate statistically significant relative risk, but no statistically significant relative risks were ultimately found.
- We had initially planned to use the Paule-Mandel variance estimator for the random-effect model instead of the DerSimonian-Laird one, according to appropriate guidance at the time of protocol writing. However, more recent guidance (from the same group of the first guidance) subsequently suggested a REML approach as a more appropriate and therefore this was ultimately chosen.
- Possible sources of heterogeneity were planned a priori in the protocol to be sought through mixed-effects subgroup analyses and random-effects meta-regression for meta-analyses of at least five studies. This could ultimately not be assessed, as less than 5 studies were included in any meta-analysis.
- Reporting biases were planned to be assessed for meta-analyses of at least 10 studies using contour-enhanced funnel plots and with the Egger's weighted regression test. This could ultimately not be assessed, as less than 10 studies were included in any meta-analysis.
- The robustness of the results was planned to be checked a priori with sensitivity analyses based on (i) inclusion/exclusion of trials with low risk of bias and (ii) improvement of the GRADE classification. However, all results were based on one, two, or seldom three trials and therefore any trial omissions were not deemed stable.

Appendix 3. List of studies identified from the literature and their inclusion/exclusion status, with reasons.

Nr	Paper	Status
Exclusion by title / abstract		
1	{NCT00519415}. Comparative Results of Maxillary Deficiency Treatment by Tongue Plate and Facemask in Growing Patients. Completed	Excluded by title
2	{NCT01770782}. Orthodontic Retention on the Maxillary Stability After SARME Using Laser Scanner. Completed	Excluded by title
3	{NCT02179593}. Effectiveness of SARPE With 3 and 2-Segment Technique: A Randomized Clinical Trial. Active, not recruiting	Excluded by title
4	{NCT02574117}. Cone Beam Computed Tomography for Evaluating Corticotomy-assisted Maxillary Expansion. Completed	Excluded by title
5	{NCT03267927}. Management of Obstructive Sleep Apnea (OSA) in Children and Maxillary and Mandibular Development. Recruiting	Excluded by title
6	Adolphs N, Ernst N, Menneking H, Hoffmeister B. Transpalatal distraction--state of the art for the individual management of transverse maxillary deficiency--a review of 50 consecutive cases. J Craniomaxillofac Surg. 2014;42(8):1669-74.	Excluded by title
7	Aglarci C, Esenlik E, Findik Y. Comparison of short-term effects between face mask and skeletal anchorage therapy with intermaxillary elastics in patients with maxillary retrognathia. Eur J Orthod 2016;38(3):313-23.	Excluded by title
8	Akay MC, Aras I, Gunbay T, Aras A. Does transpalatal distraction affect pharyngeal airway dimensions and related soft tissues? J Oral Maxillofac Surg. 2014;72(8):1559-64.	Excluded by title
9	Akay MC. IS THE MIDLINE BUCCAL INCISION IMPORTANT FOR THE OUTCOME OF SURGICALLY ASSISTED RAPID MAXILLARY EXPANSION? In reply. Journal of Oral and Maxillofacial Surgery. 2010;68(11):2924-.	Excluded by title
10	Akis H, Doruk C. Dentofacial Effects of Fixed Functional Appliances with or without Mini Screw Anchorage in the Treatment of Class II Division I Malocclusion: A Finite Element Analysis. Turkish Journal of Orthodontics. 2018;31(1):7-12.	Excluded by title
11	Alyessary AS, Yap AU, Othman SA, Rahman MT, Al-Namnam NM, Radzi Z. Is there an optimal initial amount of activation for midpalatal suture expansion? : A histomorphometric and immunohistochemical study in a rabbit model. J Orofac Orthop. 2018;79(3):169-79.	Excluded by title
12	Alyessary AS, Yap AUJ, Othman SA, Ibrahim N, Rahman MT, Radzi Z. Bone-borne accelerated sutural expansion: A microcomputed tomography study in rabbits. American Journal of Orthodontics and Dentofacial Orthopedics. 2018;154(2):260-9.	Excluded by title
13	Alyessary AS, Yap AUJ, Othman SA, Rahman MT, Radzi Z. Effect of Piezoelectric Sutural Ostectomies on Accelerated Bone-Borne Sutural Expansion. J Oral Maxillofac Surg. 2018;76(3):616-30.	Excluded by title
14	Alyessary AS, Yap AUJ, Othman SA, Rahman MT, Radzi Z. Effect of Piezoelectric Sutural Ostectomies on Accelerated Bone-Borne Sutural Expansion. Journal of Oral and Maxillofacial Surgery. 2017.	Excluded by title
15	Angelieri F, Ruellas AC, Yatabe MS, Cevidanes LHS, Franchi L, Toyama-Hino C, et al. Zygomaticomaxillary suture maturation: Part IIThe influence of sutural maturation on the response to maxillary protraction. Orthodontics & Craniofacial Research. 2017;20(3):152-63.	Excluded by title
16	Asscherickx K, Govaerts E, Aerts J, Vande Vannet B. Maxillary changes with bone-borne surgically assisted rapid palatal expansion: A prospective study. Am J Orthod Dentofacial Orthop. 2016;149(3):374-83.	Excluded by title
17	Aziz SR, Tanchyk A. Surgically assisted palatal expansion with a bone-borne self-retaining palatal expander. J Oral Maxillofac Surg. 2008;66(9):1788-93.	Excluded by title
18	Baccetti T, Clerck HJ, Cevidanes LH, Franchi L. Morphometric analysis of treatment effects of bone-anchored maxillary protraction in growing Class III patients. Eur J Orthod 2011;33(2):121-5.	Excluded by title
19	Baek SH, Kim KW, Choi JY. New treatment modality for maxillary hypoplasia in cleft patients Protraction facemask with miniplate anchorage. Angle Orthod 2010;80(4):783-91.	Excluded by title
20	Baek SH, Park YH, Chung JH, Kim S, Choi JY. Orthodontic and orthopedic treatment for a growing patient with Tessier number 0 cleft. Korean Journal of Orthodontics. 2018;48(2):113-24.	Excluded by title
21	Baek SH, Seo YJ. Application of orthodontic mini-implants and ligation for absolute skeletal anchorage to the intraoral labiolingual appliance: midface distraction osteogenesis cases treated with the RED System. J Craniofac Surg. 2011;22(2):609-13.	Excluded by title
22	Baik UB, Chun YS, Jung MH, Sugawara J. Protraction of mandibular second and third molars into missing first molar spaces for a patient with an anterior open bite and anterior spacing. Am J Orthod Dentofacial Orthop 2012;141(6):783-95.	Excluded by title
23	Batra P, Agrawal V, Kiran HJ, Madanagowda SB. Treatment of a patient with a bilateral cleft lip and palate with implants and surgery of the maxillary anterior region. World J Orthod. 2010;11(4):380-6.	Excluded by title
24	Battistetti GD, Sinegalia AC, Fleig CdN, Bombonatti R. Nova proposta de expansor maxilar com ancoragem óssea: relato de caso clínico. Rev Clín Ortod Dent Press. 2011;10(1):28-34.	Excluded by title
25	Bicalho RdF, Bicalho JS, Laboissiere Jr M. Utilização de microparafuso ortodôntico autoperfurante para reabilitação temporária de incisivo lateral superior. ImplantNews. 2010;7(3):389-96.	Excluded by title
26	Bila M, Defrancq J, Nadjmi N, Renier L, Stevens S, Vanassche B, et al. CBCT analysis of skeletal, dental and nasal changes after transpalatal distraction. International Journal of Oral and Maxillofacial Surgery. 2015;44:e39.	Excluded by title
27	Block MS, Akin R, Chang A, Gottsegen GB, Gardiner D. Skeletal and dental movements after anterior maxillary advancement using implant-supported distraction osteogenesis in dogs. Journal of Oral and Maxillofacial Surgery. 1997;55(12):1433-9.	Excluded by title
28	Bozkaya E, Yuksel AS, Bozkaya S. Zygomatic miniplates for skeletal anchorage in orthopedic correction of Class 111 malocclusion: A controlled clinical trial. Korean J Orthod 2017;47(2):118-29.	Excluded by title
29	Buzatta LN, Toyofuku ACMM, Pinto AdS, Gandini Junior LG, Shimizu RH. Expansão rápida da maxila osseossuportada: um relato de caso e passo a passo laboratorial. Ortho Sci, Orthod sci pract. 2017;10(39):223-33.	Excluded by title
30	Byloff FK, Mossaz CF. Skeletal and dental changes following surgically assisted rapid palatal expansion. Eur J Orthod 2004;26(4):403-9.	Excluded by title
31	Cai M, Shen GF. [Development of bone-anchored surgically assisted rapid maxillary expansion]. Shanghai Kou Qiang Yi Xue. 2009;18(4):441-5.	Excluded by title
32	Camps-Pereperez I, Guijarro-Martinez R, Peiro-Guijarro MA, Hernandez-Alfaro F. The value of cone beam computed tomography imaging in surgically assisted rapid palatal expansion: a systematic review of the literature. International Journal of Oral and Maxillofacial Surgery. 2017;46(7):827-38.	Excluded by title
33	Carlino F, Cortese A. Original technique for mandibular transversal surgical expansion via a tooth-borne lingual device. International Journal of Oral and Maxillofacial Surgery. 2015;44:e49.	Excluded by title
34	Carlino F, Pantaleo G, Ciuffolo F, Claudio PP, Cortese A. New Technique for Mandibular Symphyseal Distraction by a Double-Level Anchorage and Fixation System: Advantages and Results. J Craniofac Surg. 2016;27(6):1469-75.	Excluded by title
35	Carneiro JT, Jr., Paschoal EH, Carreira AS, Real RP. Carotid cavernous fistula after surgically assisted rapid maxillary expansion with a bone anchored appliance. Int J Oral Maxillofac Surg. 2013;42(3):326-8.	Excluded by title
36	Carpentier S, van Gastel J, Schoenaers J, Carels C, Vander Poorten V, Coucke W, et al. Evaluation of Transverse Maxillary Expansion After a Segmental Posterior Subapical Maxillary Osteotomy in Cleft Lip and Palate Patients With Severe Collapse of the Lateral Maxillary Segments. Cleft Palate-Craniofacial Journal. 2014;51(6):651-7.	Excluded by title
37	Carvalho Trojan L, Andres Gonzalez-Torres L, Claudia Moreira Melo A, Barbosa de Las Casas E. Stresses and Strains Analysis Using Different Palatal Expander Appliances in Upper Jaw and Midpalatal Suture. Artif Organs. 2017;41(6):E41-e51.	Excluded by title
38	Cevidanes L, Baccetti T, Franchi L, McNamara JA, Jr., De Clerck H. Comparison of two protocols for maxillary protraction: bone anchors versus face mask with rapid maxillary expansion. Angle Orthod. 2010;80(5):799-806.	Excluded by title
39	Cha BK, Choi DS, Ngan P, Jost-Brinkmann PG, Kim SM, Jang IS. Maxillary protraction with miniplates providing skeletal anchorage in a growing Class III patient. Am J Orthod Dentofacial Orthop. 2011;139(1):99-112.	Excluded by title
40	Choi SH, Kang DY, Hwang CJ. Adult patient with hemifacial microsomia treated with combined orthodontics and distraction osteogenesis. Am J Orthod Dentofacial Orthop. 2014;145(1):72-84.	Excluded by title
41	Cortese A, de Cristofaro M, Savastano G. A new transpalatal distraction device: Report of three cases with surgical and occlusal evaluations. Proceedings of the 7th European Craniofacial Congress2003. p. 57-67.	Excluded by title
42	Cortese A, Savastano G, Savastano M, Spagnuolo G, Papa F. New Technique: Le Fort I Osteotomy for Maxillary Advancement and Palatal Distraction in 1 Stage. Journal of Oral and Maxillofacial Surgery. 2009;67(1):223-8.	Excluded by title
43	Cousley RR. A clinical strategy for maxillary molar intrusion using orthodontic mini-implants and a customized palatal arch. J Orthod.	Excluded by title

	2010;37(3):202-8.	
44	Cousley RR. Molar intrusion in the management of anterior openbite and 'high angle' Class II malocclusions. J Orthod. 2014;41 Suppl 1:S39-46.	Excluded by title
45	Cousley RRJ. Molar intrusion in the management of anterior openbite and 'high angle' Class II malocclusions. Journal of Orthodontics. 2014;41:S39-S46.	Excluded by title
46	Cunha ACD, Lee H, Nojima LI, Nojima M, Lee KJ. Miniscrew-assisted rapid palatal expansion for managing arch perimeter in an adult patient. Dental Press J Orthod. 2017;22(3):97-108.	Excluded by title
47	De Clerck EEB, Swennen GRJ. Success rate of miniplate anchorage for bone anchored maxillary protraction. Angle Orthod 2011;81(6):1010-3.	Excluded by title
48	De Clerck H, Nguyen T, de Paula LK, Cevidanes L. Three-dimensional assessment of mandibular and glenoid fossa changes after bone-anchored Class III intermaxillary traction. Am J Orthod Dentofacial Orthop 2012;142(1):25-31.	Excluded by title
49	de Gijt JP, Gul A, Sutedja H, Wolvius EB, van der Wal KG, Koudstaal MJ. Long-term (6.5 years) follow-up of mandibular midline distraction. J Craniomaxillofac Surg. 2016;44(10):1576-82.	Excluded by title
50	de Gijt JP, Gul A, Tjoa STH, Wolvius EB, van der Wal KGH, Koudstaal MJ. Follow up of surgically-assisted rapid maxillary expansion after 6.5 years: skeletal and dental effects. British Journal of Oral & Maxillofacial Surgery. 2017;55(1):56-60.	Excluded by title
51	de Menezes LM, de Oliveira RB, Weissheimer A, Avelar RL. Midfacial Protraction With Skeletal Anchorage After Pterygomaxillary Separation. J Craniofac Surg. 2016;27(6):1561-4.	Excluded by title
52	Deeb W, Hansen L, Hotan T, Hietschold V, Harzer W, Tausche E. Changes in nasal volume after surgically assisted bone-borne rapid maxillary expansion. Am J Orthod Dentofacial Orthop. 2010;137(6):782-9.	Excluded by title
53	Defrancq E, Weckx K, Nadjmi N, Van HG, Vercruysse H, Van Der Dussen N, et al. Cone Beam CT assessment of nasal septal deviation changes following surgical rapid maxillary expansion. Xx Congress of the European Association of Cranio-Maxillo-Facial-Surgery. 2010:101-12.	Excluded by title
54	Di Leonardo B, Ludwig B, Lisson JA, Contardo L, Mura R, Hourfar J. Insertion torque values and success rates for paramedian insertion of orthodontic mini-implants. Journal of Orofacial Orthopedics-Fortschritte Der Kieferorthopadie. 2018;79(2):109-15.	Excluded by title
55	Eid OM, Ramadan AAF, Nadim MA, Hamed TAB. Maxillary protraction using orthodontic miniplates in correction of Class III malocclusion during growth. Journal of the World Federation of Orthodontists. 2016;5(3):100-6.	Excluded by title
56	Elnagar MH, Elshourbagy E, Ghobashy S, Khedr M, Evans CA. Comparative evaluation of 2 skeletally anchored maxillary protraction protocols. Am J Orthod Dentofacial Orthop. 2016;150(5):751-62.	Excluded by title
57	Elnagar MH, Elshourbagy E, Ghobashy S, Khedr M, Evans CA. Dentoalveolar and arch dimension changes in patients treated with miniplate-anchored maxillary protraction. Am J Orthod Dentofacial Orthop. 2017;151(6):1092-106.	Excluded by title
58	Elnagar MH, Elshourbagy E, Ghobashy S, Khedr M, Kusnoto B, Evans CA. Three-dimensional assessment of soft tissue changes associated with bone-anchored maxillary protraction protocols. Am J Orthod Dentofacial Orthop. 2017;152(3):336-47.	Excluded by title
59	El-Sayed KM, Khalil H. Transpalatal distraction osteogenesis prior to alveolar bone grafting in cleft lip and palate patients. Int J Oral Maxillofac Surg. 2010;39(8):761-6.	Excluded by title
60	El-Sayed KM, Khalil H. Transpalatal distraction osteogenesis prior to alveolar bone grafting in cleft lip and palate patients. International Journal of Oral and Maxillofacial Surgery. 2010;39(8):761-6.	Excluded by title
61	Evans CA, Scarfe WC, Ahmad M, Cevidanes LHS, Ludlow JB, Palomo JM, et al. Clinical recommendations regarding use of cone beam computed tomography in orthodontics. Position statement by the American Academy of Oral and Maxillofacial Radiology. Oral Surgery Oral Medicine Oral Pathology Oral Radiology. 2013;116(2):238-57.	Excluded by title
62	Feng XX, Li JH, Li Y, Zhao ZH, Zhao S, Wang J. Effectiveness of TAD-anchored maxillary protraction in late mixed dentition A systematic review. Angle Orthod 2012;82(6):1107-14.	Excluded by title
63	Finn MD. Surgical Assistance for Rapid Orthodontic Treatment and Temporary Skeletal Anchorage. Oral and Maxillofacial Surgery Clinics of North America. 2014;26(4):539-+.	Excluded by title
64	Finn MD. Surgical assistance for rapid orthodontic treatment and temporary skeletal anchorage. Oral Maxillofac Surg Clin North Am. 2014;26(4):539-50.	Excluded by title
65	Fukui T, Fukui K, Yoshida K, Tsuruta M, Galang-Boquiren MTS. Invisible treatment of a severe Class II deep over bite with narrow mandibular dental arch with multilingual bracket appliances. Journal of the World Federation of Orthodontists. 2017;6(2):69-79.	Excluded by title
66	Gamage SN, Goss AN. Surgically-assisted rapid maxillary expansion of narrowed maxillae: a case-cohort study. Australian Orthodontic Journal. 2013;29(1):21-7.	Excluded by title
67	Gamble J, Lagravere MO, Major PW, Heo G. New statistical method to analyze three-dimensional landmark configurations obtained with cone-beam CT: basic features and clinical application for rapid maxillary expansion. Korean J Radiol. 2012;13(2):126-35.	Excluded by title
68	Gamble J, Lagravère MO, Major PW, Heo G. New statistical method to analyze three-dimensional landmark configurations obtained with cone-beam CT: Basic features and clinical application for rapid maxillary expansion. Korean Journal of Radiology. 2012;13(2):126-35.	Excluded by title
69	Garib D, Yatabe M, de Souza Faco RA, Gregorio L, Cevidanes L, de Clerck H. Bone-anchored maxillary protraction in a patient with complete cleft lip and palate: A case report. Am J Orthod Dentofacial Orthop. 2018;153(2):290-7.	Excluded by title
70	Garreau E, Bouscaillou J, Rattier S, Ferri J, Raoul G. Bone-borne distractor versus tooth-borne distractor for orthodontic distraction after surgical maxillary expansion: The patient's point of view. Int Orthod. 2016;14(2):214-32.	Excluded by title
71	Garreau E, Wojcik T, Rakotomalala H, Raoul G, Ferri J. Symphyseal distraction in the context of orthodontic treatment: A series of 35 cases. International Orthodontics. 2015;13(1):81-95.	Excluded by title
72	Gautam P, Zhao LP, Patel P. Determining the osteotomy pattern in surgically assisted rapid maxillary expansion in a unilateral palatal cleft A finite element model approach. Angle Orthod 2011;81(3):410-9.	Excluded by title
73	Ge YS, Liu J, Chen L, Han JL, Guo X. Dentofacial effects of two facemask therapies for maxillary protraction Miniscrew implants versus rapid maxillary expanders. Angle Orthodontist. 2012;82(6):1083-91.	Excluded by title
74	Ge YS, Liu J, Chen L, Han JL, Guo X. Dentofacial effects of two facemask therapies for maxillary protraction. Angle Orthod. 2012;82(6):1083-91.	Excluded by title
75	Gerlach KL, Zahl C. Surgically assisted rapid palatal expansion using a new distraction device: Report of a case with an epimucosal fixation. Journal of Oral and Maxillofacial Surgery. 2005;63(5):711-3.	Excluded by title
76	Goonewardene M, Allan B. Biomechanics and Treatment of Dentofacial Deformity. Biomechanical Foundation of Clinical Orthodontics2015. p. 389-432.	Excluded by title
77	Gunbay T, Akay MC, Aras A, Gomel M. Effects of transmandibular symphyseal distraction on teeth, bone, and temporomandibular joint. J Oral Maxillofac Surg. 2009;67(10):2254-65.	Excluded by title
78	Gunbay T, Akay MC, Aras A, Gomel M. Effects of Transmandibular Symphyseal Distraction on Teeth, Bone, and Temporomandibular Joint. Journal of Oral and Maxillofacial Surgery. 2009;67(10):2254-65.	Excluded by title
79	Hamedi-Sangsari A, Chinipardaz Z, Carrasco L. Following Surgically Assisted Rapid Palatal Expansion, Do Tooth-Borne or Bone-Borne Appliances Provide More Skeletal Expansion and Dental Expansion? J Oral Maxillofac Surg. 2017;75(10):2211-22.	Excluded by title
80	Hernández-Alfaro F, Guijarro-Martínez R, Peiró-Guijarro MA. Surgery first in orthognathic surgery: What have we learned? A comprehensive workflow based on 45 consecutive cases. Journal of Oral and Maxillofacial Surgery. 2014;72(2):376-90.	Excluded by title
81	Hernandez-Alfaro F, Mareque Bueno J, Diaz A, Pages CM. Minimally invasive surgically assisted rapid palatal expansion with limited approach under sedation: a report of 283 consecutive cases. J Oral Maxillofac Surg. 2010;68(9):2154-8.	Excluded by title
82	Hess D, Buser D, Dietschi D, Grossen G, Schonenberger A, Belzer UC. Esthetic single-tooth replacement with implants: a team approach. Quintessence Int. 1998;29(2):77-86.	Excluded by title
83	Heymann GC, Cevidanes L, Cornelis M, De Clerck HJ, Tulloch JFC. Three-dimensional analysis of maxillary protraction with intermaxillary elastics to miniplates. Am J Orthod Dentofacial Orthop 2010;137(2):274-84.	Excluded by title
84	Hino CT, Cevidanes LH, Nguyen TT, De Clerck HJ, Franchi L, McNamara JA, Jr. Three-dimensional analysis of maxillary changes associated with facemask and rapid maxillary expansion compared with bone anchored maxillary protraction. Am J Orthod Dentofacial Orthop. 2013;144(5):705-14.	Excluded by title
85	Hino CT, Cevidanes LHS, Nguyen TT, De Clerck HJ, Franchi L, McNamara Jr JA. Three-dimensional analysis of maxillary changes associated with facemask and rapid maxillary expansion compared with bone anchored maxillary protraction. American Journal of	Excluded by title

	Orthodontics and Dentofacial Orthopedics. 2013;144(5):705-14.	
86	Holty JEC, Guilleminault C. Surgical Options for the Treatment of Obstructive Sleep Apnea. Medical Clinics of North America. 2010;94(3):479-515.	Excluded by title
87	Hourfar J, Kinzinger GS, Ludwig B, Spindler J, Lisson JA. Differential treatment effects of two anchorage systems for rapid maxillary expansion: a retrospective cephalometric study. J Orofac Orthop. 2016;77(5):314-24.	Excluded by title
88	Hourfar J, Kinzinger GSM, Ludwig B, Spindler J, Lisson JA. Differential treatment effects of two anchorage systems for rapid maxillary expansion: a retrospective cephalometric study. Journal of Orofacial Orthopedics. 2016;77(5):314-24.	Excluded by title
89	Huizinga MP, Meulstee JW, Dijkstra PU, Schepers RH, Jansma J. Bone-borne surgically assisted rapid maxillary expansion: A retrospective three-dimensional evaluation of the asymmetry in expansion. Journal of Cranio-Maxillofacial Surgery. 2018;46(8):1329-35.	Excluded by title
90	Iglesia Gdl, Walter A, Iglesia Fdl, Winsauer H, Puigdollers A. Stability of the anterior arm of three different Hyrax hybrid expanders: an in vitro study. Dental Press J Orthod. 2018;23(1):37-45.	Excluded by title
91	Ito Y, Kawamoto T, Moriyama K. The orthopaedic effects of bone-anchored maxillary protraction in a beagle model. Eur J Orthod 2014;36(6):632-40.	Excluded by title
92	Jahanbin A, Kazemian M, Eslami N, Pouya IS. Maxillary Protraction With Intermaxillary Elastics to Miniplates Versus Bone-Anchored Face-Mask Therapy in Cleft Lip and Palate Patients. J Craniofac Surg. 2016;27(5):1247-52.	Excluded by title
93	Jahanbin A, Kazemian M, Eslami N, Pouya IS. Maxillary Protraction With Intermaxillary Elastics to Miniplates Versus Bone-Anchored Face-Mask Therapy in Cleft Lip and Palate Patients. Journal of Craniofacial Surgery. 2016;27(5):1247-52.	Excluded by title
94	Jensen T, Rodrigo-Domingo M. Surgically assisted rapid maxillary expansion (SARME) with or without intraoperative releasing of the nasal septum. Oral Surgery Oral Medicine Oral Pathology Oral Radiology. 2017;123(3):E85-E90.	Excluded by title
95	Jeon YJ, Kim YH, Son WS, Hans MG. Correction of a canted occlusal plane with miniscrews in a patient with facial asymmetry. Am J Orthod Dentofacial Orthop. 2006;130(2):244-52.	Excluded by title
96	Jeon YJ, Kim YH, Son WS, Hans MG. Correction of a canted occlusal plane with miniscrews in a patient with facial asymmetry. American Journal of Orthodontics and Dentofacial Orthopedics. 2006;130(2):244-52.	Excluded by title
97	Jung SK, Kim TW. Treatment of unilateral posterior crossbite with facial asymmetry in a female patient with transverse discrepancy. Am J Orthod Dentofacial Orthop 2015;148(1):154-64.	Excluded by title
98	Kale B, Buyukcavus MH. Pure skeletal maxillary protraction with skeletal anchorage in high-angle Class III patients: A case series. Journal of the World Federation of Orthodontists. 2018;7(2):66-78.	Excluded by title
99	Kang YG, Kim JY, Nam JH. Control of maxillary dentition with 2 midpalatal orthodontic miniscrews. American Journal of Orthodontics and Dentofacial Orthopedics. 2011;140(6):879-85.	Excluded by title
100	Karamanli BD, Kilicoglu H, Karamanli AF. Evaluation of the Effects of the Dental and Skeletal Anchored Face Mask Therapies on the Craniofacial System by Using Nonlinear Finite Element Analysis. Apos Trends in Orthodontics. 2017;7(6):267-72.	Excluded by title
101	Karydis A, Trojan T, Vassar JW, Fisher J. Re: Response to: Dentoskeletal effects of a temporary skeletal anchorage device-supported rapid maxillary expansion appliance (TSADRME): A pilot study. Vassar JW, Karydis A, Trojan T, Fisher J. The Angle Orthod 2016;86:241-249. Angle Orthod 2017;87(4):636-.	Excluded by title
102	Kayalar E, Schauseil M, Kuvat SV, Emekli U, Firatli S. Comparison of tooth-borne and hybrid devices in surgically assisted rapid maxillary expansion: A randomized clinical cone-beam computed tomography study. Journal of Cranio-Maxillofacial Surgery. 2016;44(3):285-93.	Excluded by title
103	Kircelli BH, Pektas ZO, Uckan S. Orthopedic protraction with skeletal anchorage in a patient with maxillary hypoplasia and hypodontia. Angle Orthod. 2006;76(1):156-63.	Excluded by title
104	Kircelli BH, Pektaş ZO, Uçkan S. Orthopedic protraction with skeletal anchorage in a patient with maxillary hypoplasia and hypodontia. Angle Orthodontist. 2006;76(1):156-63.	Excluded by title
105	Kircelli BH, Pektas ZO. Midfacial protraction with skeletally anchored face mask therapy: a novel approach and preliminary results. Am J Orthod Dentofacial Orthop. 2008;133(3):440-9.	Excluded by title
106	Kircelli BH, Pektas ZO. Midfacial protraction with skeletally anchored face mask therapy: A novel approach and preliminary results. American Journal of Orthodontics and Dentofacial Orthopedics. 2008;133(3):440-9.	Excluded by title
107	Kobayashi M, Fushima K. Orthodontic skeletal anchorage using a palatal external plate. J Orthod. 2014;41(1):53-62; quiz 3-4.	Excluded by title
108	Koudstaal MJ, Poort LJ, van der Wal KG, Wolvius EB, Prahl-Andersen B, Schulten AJ. Surgically assisted rapid maxillary expansion (SARME): a review of the literature. Int J Oral Maxillofac Surg. 2005;34(7):709-14.	Excluded by title
109	Koudstaal MJ, Poort LJ, van der Wal KGH, Wolvius EB, Prahl-Andersen B, Schulten AJM. Surgically assisted rapid maxillary expansion (SARME): A review of the literature. International Journal of Oral and Maxillofacial Surgery. 2006;34(7):709-14.	Excluded by title
110	Koudstaal MJ, Smeets JB, Kleinrensink GJ, Schulten AJ, van der Wal KG. Relapse and stability of surgically assisted rapid maxillary expansion: an anatomic biomechanical study. J Oral Maxillofac Surg. 2009;67(1):10-4.	Excluded by title
111	Koudstaal MJ, Smeets JBJ, Kleinrensink GJ, Schulten AJM, van der Wal KGH. Relapse and Stability of Surgically Assisted Rapid Maxillary Expansion: An Anatomic Biomechanical Study. Journal of Oral and Maxillofacial Surgery. 2009;67(1):10-4.	Excluded by title
112	Koudstaal MJ, van der Wal KGH, Wolvius EB. Experience with the transpalatal distractor in congenital deformities. Mund - Kiefer - und Gesichtschirurgie. 2006;10(5):331-4.	Excluded by title
113	Koudstaal MJ, Wolvius EB, Schulten AJ, Hop WC, van der Wal KG. Stability, tipping and relapse of bone-borne versus tooth-borne surgically assisted rapid maxillary expansion; a prospective randomized patient trial. Int J Oral Maxillofac Surg. 2009;38(4):308-15.	Excluded by title
114	Kunz F, Linz C, Baunach G, Bohm H, Meyer-Marcotty P. Expansion patterns in surgically assisted rapid maxillary expansion : Transpalatal distractor versus hyrax appliance. J Orofac Orthop. 2016;77(5):357-65.	Excluded by title
115	Kwon HY, Mah SJ, Kang YG. Asymmetric transverse control of maxillary dentition with two midpalatal orthodontic miniscrews. Angle Orthod. 2015;85(3):525-34.	Excluded by title
116	Kyung SH, Hong SG, Park YC. Distalization of maxillary molars with a midpalatal miniscrew. J Clin Orthod. 2003;37(1):22-6.	Excluded by title
117	Landes CA, Laudemann K, Petruchin O, Mack MG, Kopp S, Ludwig B, et al. Comparison of bipartite versus tripartite osteotomy for maxillary transversal expansion using 3-dimensional preoperative and postexpansion computed tomography data. J Oral Maxillofac Surg. 2009;67(10):2287-301.	Excluded by title
118	Landes CA, Laudemann K, Schubel F, Petruchin O, Mack M, Kopp S, et al. Comparison of tooth- and bone-borne devices in surgically assisted rapid maxillary expansion by three-dimensional computed tomography monitoring: transverse dental and skeletal maxillary expansion, segmental inclination, dental tipping, and vestibular bone resorption. J Craniofac Surg. 2009;20(4):1132-41.	Excluded by title
119	Laudemann K, Petruchin O, Mack MG, Kopp S, Sader R, Landes CA. Evaluation of surgically assisted rapid maxillary expansion with or without pterygomaxillary disjunction based upon preoperative and post-expansion 3D computed tomography data. Oral Maxillofac Surg. 2009;13(3):159-69.	Excluded by title
120	Laudemann K, Petruchin O, Nafzger M, Ballon A, Kopp S, Sader RA, et al. Long-term 3D cast model study: bone-borne vs. tooth-borne surgically assisted rapid maxillary expansion due to secondary variables. Oral Maxillofac Surg. 2010;14(2):105-14.	Excluded by title
121	Laudemann K, Santo G, Revilla C, Harth M, Kopp S, Sader RA, et al. Assessment of surgically assisted rapid maxillary expansion regarding pterygomaxillary disjunction using thin volume-rendering technique: in variance analysis and in reliability, accuracy, and validity. J Oral Maxillofac Surg. 2011;69(10):2631-43.	Excluded by title
122	Lee HK, Bayome M, Ahn CS, Kim SH, Kim KB, Mo SS, et al. Stress distribution and displacement by different bone-borne palatal expanders with micro-implants: A three-dimensional finite-element analysis. European Journal of Orthodontics. 2014;36(5):531-40.	Excluded by title
123	Lee JH, Choo H, Kim SH, Chung KR, Giannuzzi LA, Ngan P. Replacing a failed mini-implant with a miniplate to prevent interruption during orthodontic treatment. Am J Orthod Dentofacial Orthop. 2011;139(6):849-57.	Excluded by title
124	Lee KJ, Park YC, Park JY, Hwang WS. Miniscrew-assisted nonsurgical palatal expansion before orthognathic surgery for a patient with severe mandibular prognathism. Am J Orthod Dentofacial Orthop. 2010;137(6):830-9.	Excluded by title
125	Lee KJ, Park YC, Park JY, Hwang WS. Miniscrew-assisted nonsurgical palatal expansion before orthognathic surgery for a patient with severe mandibular prognathism. American Journal of Orthodontics and Dentofacial Orthopedics. 2010;137(6):830-9.	Excluded by title
126	Lee SC, Park JH, Bayome M, Kim KB, Araujo EA, Kook YA. Effect of bone-borne rapid maxillary expanders with and without surgical assistance on the craniofacial structures using finite element analysis. American Journal of Orthodontics and Dentofacial Orthopedics. 2014;145(5):638-48.	Excluded by title
127	Leyder P, Wycisk G, Quilichini J. [An adjustable distractor for transverse maxillary distraction osteogenesis]. Rev Stomatol Chir Maxillofac	Excluded by title

	Chir Orale. 2013;114(3):170-4.	
128	Leyder P, Wycisk G, Quilichini J. An adjustable distractor for transverse maxillary distraction osteogenesis. Revue de Stomatologie, de Chirurgie Maxillo-faciale et de Chirurgie Orale. 2013;114(3):170-4.	Excluded by title
129	Lim SM, Hong RK. Distal movement of maxillary molars using a lever-arm and mini-implant system. Angle Orthod. 2008;78(1):167-75.	Excluded by title
130	Lim SM, Hong RK. Distal movement of maxillary molars using a lever-arm and mini-implant system. Angle Orthodontist. 2008;78(1):167-75.	Excluded by title
131	Liu SSY, Kyung HM, Buschang PH. Continuous forces are more effective than intermittent forces in expanding sutures. Eur J Orthod 2010;32(4):371-80.	Excluded by title
132	Liu SSY, Opperman LA, Kyung HM, Buschang PH. Is there an optimal force level for sutural expansion? Am J Orthod Dentofacial Orthop 2011;139(4):446-55.	Excluded by title
133	Liu SSY, Xu HS, Sun J, Kontogiorgos E, Whittington PR, Misner KG, et al. Recombinant human bone morphogenetic protein-2 stimulates bone formation during interfrontal suture expansion in rabbits. Am J Orthod Dentofacial Orthop 2013;144(2):210-7.	Excluded by title
134	Liu SY, Guilleminault C, Huon LK, Yoon A. Distraction Osteogenesis Maxillary Expansion (DOME) for Adult Obstructive Sleep Apnea Patients with High Arched Palate. Otolaryngol Head Neck Surg. 2017;157(2):345-8.	Excluded by title
135	Lombardo L, Carlucci A, Maino BG, Colonna A, Paoletto E, Siciliani G. Class III malocclusion and bilateral cross-bite in an adult patient treated with miniscrew-assisted rapid palatal expander and aligners. Angle Orthodontist. 2018;88(5):649-64.	Excluded by title
136	Ludwig B, Baumgaertel S, Zorkun B, Bonitz L, Glasl B, Wilmes B, et al. Application of a new viscoelastic finite element method model and analysis of miniscrew-supported hybrid hyrax treatment. Am J Orthod Dentofacial Orthop 2013;143(3):426-35.	Excluded by title
137	Ludwig B, Glas B, Bowman SJ, Drescher D, Wilmes B. Miniscrew-supported Class III treatment with the Hybrid RPE Advancer. J Clin Orthod. 2010;44(9):533-9; quiz 61.	Excluded by title
138	Lui DW, Chou J, Carrasco L. Nasolabial volumetric symmetry following sarpe. Journal of Oral and Maxillofacial Surgery. 2011;69(9):e27-e8.	Excluded by title
139	Ma QL, Conley RS, Wu T, Li H. Interdisciplinary treatment for an adult with a unilateral cleft lip and palate. Am J Orthod Dentofacial Orthop. 2014;146(2):238-48.	Excluded by title
140	Ma QL, Conley RS, Wu T, Li H. Interdisciplinary treatment for an adult with a unilateral cleft lip and palate. American Journal of Orthodontics and Dentofacial Orthopedics. 2014;146(2):238-48.	Excluded by title
141	Madhavarajan S, Taylor N, Johnson P. Surgically assisted rapid palatal expansion (SARPE). British Journal of Oral and Maxillofacial Surgery. 2014;52(8):e59-e60.	Excluded by title
142	Maino G, Turci Y, Arreghini A, Paoletto E, Siciliani G, Lombardo L. Skeletal and dentoalveolar effects of hybrid rapid palatal expansion and facemask treatment in growing skeletal Class III patients. Am J Orthod Dentofacial Orthop. 2018;153(2):262-8.	Excluded by title
143	Malkoc S, Usumez S, Iseri H. Long-term effects of symphyseal distraction and rapid maxillary expansion on pharyngeal airway dimensions, tongue, and hyoid position. American Journal of Orthodontics and Dentofacial Orthopedics. 2007;132(6):769-75.	Excluded by title
144	Melsen B. Have the indications for TADs increased? International Journal of Oral and Maxillofacial Surgery. 2013;42(10):1333.	Excluded by title
145	Mendes Junior TE, Lima AB, Mendes TE, Mendes CV, Rosario HD, Paranhos LR. Distalization controlled with the use of lip-bumper and mini-screw as anchorage: a new approach. Int J Orthod Milwaukee. 2015;26(1):29-32.	Excluded by title
146	Meyns J, Brasil DM, Mazzi-Chaves JF, Politis C, Jacobs R. The clinical outcome of skeletal anchorage in interceptive treatment (in growing patients) for class III malocclusion. International Journal of Oral and Maxillofacial Surgery. 2018;47(8):1003-10.	Excluded by title
147	Millwaters M, Sharma PK. The role of distraction osteogenesis in patients presenting with dento-facial deformity-An overview. Seminars in Orthodontics. 2015;21(1):46-58.	Excluded by title
148	Mitsugi M, Ito O, Alcalde RE. Maxillary bone transportation in alveolar cleft-transport distraction osteogenesis for treatment of alveolar cleft repair. Br J Plast Surg. 2005;58(5):619-25.	Excluded by title
149	Mohlhenrich SC, Modabber A, Kniha K, Peters F, Steiner T, Holzle F, et al. Simulation of three surgical techniques combined with two different bone-borne forces for surgically assisted rapid palatal expansion of the maxillofacial complex: a finite element analysis. International Journal of Oral and Maxillofacial Surgery. 2017;46(10):1306-14.	Excluded by title
150	Mommaerts MY, Collado J, Mareque Bueno J. Morbidity related to "endo-corticotomies" for transpalatal osteodistraction. J Craniomaxillofac Surg. 2008;36(4):198-202.	Excluded by title
151	Mommaerts MY. Bone anchored intraoral device for transmandibular distraction. Br J Oral Maxillofac Surg. 2001;39(1):8-12.	Excluded by title
152	Moon W. Class III treatment by combining facemask (FM) and maxillary skeletal expander (MSE). Seminars in Orthodontics. 2018;24(1):95-107.	Excluded by title
153	Mujica E, Guerrero C, Gonzalez M, Lopez P. Selective maxillary widening: Distraction osteogenesis. International Journal of Oral and Maxillofacial Surgery. 2011;40(10):1118-9.	Excluded by title
154	Nada RM, Fudalej PS, Maal TJ, Berge SJ, Mostafa YA, Kuijpers-Jagtman AM. Three-dimensional prospective evaluation of tooth-borne and bone-borne surgically assisted rapid maxillary expansion. J Craniomaxillofac Surg. 2012;40(8):757-62.	Excluded by title
155	Nada RM, van Loon B, Maal TJ, Berge SJ, Mostafa YA, Kuijpers-Jagtman AM, et al. Three-dimensional evaluation of soft tissue changes in the orofacial region after tooth-borne and bone-borne surgically assisted rapid maxillary expansion. Clin Oral Investig. 2013;17(9):2017-24.	Excluded by title
156	Nada RM, van Loon B, Schols JG, Maal TJ, de Koning MJ, Mostafa YA, et al. Volumetric changes of the nose and nasal airway 2 years after tooth-borne and bone-borne surgically assisted rapid maxillary expansion. Eur J Oral Sci. 2013;121(5):450-6.	Excluded by title
157	Nahm KY, Shin SY, Ahn HW, Kim SH, Nelson G. Gummy Smile Correction Using Lingual Orthodontics and Augmented Corticotomy in Extremely Thin Alveolar Housing. J Craniofac Surg. 2017;28(7):e599-e603.	Excluded by title
158	Nevzatoğlu S, Küçükkeleş N, Güzel Z. Long-term stability of intra-oral maxillary distraction in unilateral cleft lip and palate: a case report. Australian orthodontic journal. 2013;29(2):200-8.	Excluded by title
159	Neyt NM, Mommaerts MY, Abeloos JV, De Clercq CA, Neyt LF. Problems, obstacles and complications with transpalatal distraction in non-congenital deformities. J Craniomaxillofac Surg. 2002;30(3):139-43.	Excluded by title
160	Ngan P, Wilmes B, Drescher D, Martin C, Weaver B, Gunel E. Comparison of two maxillary protraction protocols: tooth-borne versus bone-anchored protraction facemask treatment. Prog Orthod. 2015;16:26.	Excluded by title
161	Nguyen T, Cevidanes L, Cornelis MA, Heymann G, de Paula LK, De Clerck H. Three-dimensional assessment of maxillary changes associated with bone anchored maxillary protraction. Am J Orthod Dentofacial Orthop 2011;140(6):790-8.	Excluded by title
162	Nguyen T, De Clerck H, Wilson M, Golden B. Effect of Class III bone anchor treatment on airway. Angle Orthod. 2015;85(4):591-6.	Excluded by title
163	Nguyen T, De Clerck H, Wilson M, Golden B. Effect of class III bone anchor treatment on airway. Angle Orthodontist. 2015;85(4):591-6.	Excluded by title
164	Nguyen T. 3D Planning and Treatment Outcomes of Bone Anchored Maxillary Protraction. Cone Beam Computed Tomography in Orthodontics: Indications, Insights, and Innovations2014. p. 427-36.	Excluded by title
165	Nguyen T. Bone anchored maxillary protraction Response. Am J Orthod Dentofacial Orthop 2012;141(5):531-2.	Excluded by title
166	Nie P, Zhu M, Lu XF, Fang B. Bone-anchored maxillary expansion and bilateral interoral mandibular distraction osteogenesis in adult with severe obstructive sleep apnea syndrome. J Craniofac Surg. 2013;24(3):949-52.	Excluded by title
167	Nienkemper M, Wilmes B, Franchi L, Drescher D. Effectiveness of maxillary protraction using a hybrid hyrax-facemask combination: a controlled clinical study. Angle Orthod. 2015;85(5):764-70.	Excluded by title
168	Nienkemper M, Wilmes B, Franchi L, Drescher D. Effectiveness of maxillary protraction using a hybrid hyrax-facemask combination: A controlled clinical study. Angle Orthodontist. 2015;85(5):764-70.	Excluded by title
169	Nienkemper M, Wilmes B, Pauls A, Drescher D. Maxillary Protraction Using a Hybrid Hyrax-Facemask Combination. Informationen Aus Orthodontie Und Kieferorthopaedie. 2016;48(4):221-7.	Excluded by title
170	Nienkemper M, Wilmes B, Pauls A, Drescher D. Maxillary protraction using a hybrid hyrax-facemask combination. Prog Orthod. 2013;14:5.	Excluded by title
171	Nienkemper M, Wilmes B, Pauls A, Drescher D. Maxillary protraction using a hybrid hyrax-facemask combination. Progress in Orthodontics. 2013;14(1):1-8.	Excluded by title
172	Noorollahian S, Alavi S, Shirban F. Bilateral en-masse distalization of maxillary posterior teeth with skeletal anchorage: a case report. Dental Press J Orthod. 2016;21(3):85-93.	Excluded by title
173	Nowak R, Zawiślak E. An analysis of early changes to the craniofacial skeleton induced by transpalatal distraction in a patient with maxillary constriction, based on computed tomography scans – Case report. Dental and Medical Problems. 2016;53(1):134-41.	Excluded by title
174	Nucera R, Lo Giudice A, Bellocchio AM, Spinuzza P, Caprioglio A, Perillo L, et al. Bone and cortical bone thickness of mandibular buccal shelf for mini-screw insertion in adults. Angle Orthod 2017;87(5):745-51.	Excluded by title
175	Pacurar M, Vlasa A. Orthodontic anchorage on mini-implants, beetwen myth and reality. Clujul Medical. 2017;90:S22.	Excluded by title

176	Parhiz A, Schepers S, Lambrichts I, Vrielinck L, Sun Y, Politis C. Lateral cephalometry changes after SARPE. Int J Oral Maxillofac Surg. 2011;40(7):662-71.	Excluded by title
177	Park JH, Bayome M, Zahrowski JJ, Kook YA. Displacement and stress distribution by different bone-borne palatal expanders with facemask: A 3-dimensional finite element analysis. Am J Orthod Dentofacial Orthop. 2017;151(1):105-17.	Excluded by title
178	Park YC, Lee HA, Choi NC, Kim DH. Open bite correction by intrusion of posterior teeth with miniscrews. Angle Orthod 2008;78(4):699-710.	Excluded by title
179	Peretti N, MacLeod S. Zygomaticomaxillary complex fractures: Diagnosis and treatment. Current Opinion in Otolaryngology and Head and Neck Surgery. 2017;25(4):314-9.	Excluded by title
180	Petrick S, Hothan T, Hietschold V, Schneider M, Harzer W, Tausche E. Bone density of the midpalatal suture 7 months after surgically assisted rapid palatal expansion in adults. Am J Orthod Dentofacial Orthop 2011;139(4):S109-S16.	Excluded by title
181	Petrukhina O, Laudemann K, Nafzger M, Ballon A, Kopp S, Sader RA, et al. [A long-term 3D cast model analysis focusing bone-borne vs. tooth-borne SARME]. Stomatologiia (Mosk). 2011;90(3):50-3.	Excluded by title
182	Pimentel AC, Manzi MR, Barbosa AJP, Cotrim-Ferreira FA, Carvalho PEG, de Lima GF, et al. Mini-implant screws for bone-borne anchorage: A biomechanical in vitro study comparing three diameters. International Journal of Oral and Maxillofacial Implants. 2016;31(5):1072-6.	Excluded by title
183	Pimentel AC, Manzi MR, Prado Barbosa AJ, Cotrim-Ferreira FA, Guedes Carvalho PE, de Lima GF, et al. Mini-Implant Screws for Bone-Borne Anchorage: A Biomechanical In Vitro Study Comparing Three Diameters. Int J Oral Maxillofac Implants. 2016;31(5):1072-6.	Excluded by title
184	Pinto LP, Bell WH, Chu S, Buschang PH. Simultaneous 3-Dimensional Le Fort I/Distraction Osteogenesis Technique: Positional Changes. Journal of Oral and Maxillofacial Surgery. 2009;67(1):32-9.	Excluded by title
185	Pinto PX, Mommaerts MY, Wreakes G, Jacobs W. Posterior transpalatal distraction osteogenesis. J Oral Maxillofac Surg. 2002;60(5):606.	Excluded by title
186	Pulver RJ, Campbell PM, Opperman LA, Buschang PH. Miniscrew-assisted slow expansion of mature rabbit sutures. Am J Orthod Dentofacial Orthop 2016;150(2):303-12.	Excluded by title
187	Rachmiel A, Emodi O, Gutmacher Z, Blumenfeld I, Aizenbud D. Oral and dental restoration of wide alveolar cleft using distraction osteogenesis and temporary anchorage devices. J Craniomaxillofac Surg. 2013;41(8):728-34.	Excluded by title
188	Rachmiel A, Emodi O, Gutmacher Z, Blumenfeld I, Aizenbud D. Oral and dental restoration of wide alveolar cleft using distraction osteogenesis and temporary anchorage devices. Journal of Cranio-Maxillofacial Surgery. 2013;41(8):728-34.	Excluded by title
189	Rachmiel A, Srouji S, Peled M. Alveolar ridge augmentation by distraction osteogenesis. Int J Oral Maxillofac Surg. 2001;30(6):510-7.	Excluded by title
190	Raoul G, Wojcik T, Ferri J. Outcome of mandibular symphyseal distraction osteogenesis with bone-borne devices. J Craniofac Surg. 2009;20(2):488-93.	Excluded by title
191	Roelofs J, Breuning KH, van't Spijker A, Borstlap WA, Berge SJ, Kuijpers-Jagtman AM. [Unilateral surgically assisted rapid maxillary expansion using a transpalatal distractor]. Ned Tijdschr Tandheelkd. 2010;117(2):87-91.	Excluded by title
192	Safavi SM, Mohebi S, Youssefinia S, Soheilifar S. Mini-Screw Supported Intrusion Mechanics Used For Open Bite Closure and Facial Improvement: A Case Report. Int J Orthod Milwaukee. 2015;26(4):59-64.	Excluded by title
193	Sar C, Arman-Ozcirpici A, Uckan S, Yazici AC. Comparative evaluation of maxillary protraction with or without skeletal anchorage. Am J Orthod Dentofacial Orthop. 2011;139(5):636-49.	Excluded by title
194	Sari E, Kadioglu O, Ucar C, Altug HA. Prostaglandin E2 levels in gingival crevicular fluid during tooth- and bone-borne expansion. European Journal of Orthodontics. 2010;32(3):336-41.	Excluded by title
195	Sari E, Ucar C, Ceylanoglu C. Transpalatal distraction in a patient with a narrow maxilla. Angle Orthod. 2007;77(6):1126-31.	Excluded by title
196	Sari E, Uçar C, Ceylanoglu C. Transpalatal distraction in a patient with a narrow maxilla. Angle Orthodontist. 2007;77(6):1126-31.	Excluded by title
197	Savoldelli C, Lesne V, Ciszek E, Lebeau J, Bettega G. [Symphyseal distraction: a simplified procedure]. Rev Stomatol Chir Maxillofac. 2010;111(5-6):259-69.	Excluded by title
198	Scolozzi P, Verdeja R, Herzog G, Jaques B. Maxillary expansion using transpalatal distraction in patients with unilateral cleft lip and palate. Plastic and Reconstructive Surgery. 2007;119(7):2200-5.	Excluded by title
199	Seeberger R, Abe-Nickler D, Hoffmann J, Kunzmann K, Zingler S. One-stage tooth-borne distraction versus two stage bone-borne distraction in surgically assisted maxillary expansion (SARME). Oral Surg Oral Med Oral Pathol Oral Radiol. 2015;120(6):693-8.	Excluded by title
200	Seeberger R, Kater W, Schulte-Geers M, Davids R, Freier K, Thiele O. Changes after surgically-assisted maxillary expansion (SARME) to the dentoalveolar, palatal and nasal structures by using tooth-borne distraction devices. British Journal of Oral & Maxillofacial Surgery. 2011;49(5):381-5.	Excluded by title
201	Seeberger R. Surgical Correction of Transversal Deficits in the Maxilla: Current State of Surgical Techniques for Surgically Assisted Maxillary Expansion. Informationen Aus Orthodontie Und Kieferorthopaedie. 2016;48(4):271-7.	Excluded by title
202	Seitz O, Landes CA, Philipp DJ, Sader R, Klein CM. Reliable surgically assisted rapid palatal expansion by maxillary widening device. J Craniofac Surg. 2008;19(3):846-9.	Excluded by title
203	Seong EH, Choi SH, Kim HJ, Yu HS, Park YC, Lee KJ. Evaluation of the effects of miniscrew incorporation in palatal expanders for young adults using finite element analysis. Korean Journal of Orthodontics. 2018;48(2):81-9.	Excluded by title
204	Solano-Mendoza B, Iglesias-Linares A, Yanez-Vico RM, Mendoza-Mendoza A, Alio-Sanz JJ, Solano-Reina E. Maxillary protraction at early ages. The revolution of new bone anchorage appliances. J Clin Pediatr Dent. 2012;37(2):219-29.	Excluded by title
205	Stucki-McCormick SU. Distraction osteogenesis for the cleft patient-a 25-year perspective. International Journal of Oral and Maxillofacial Surgery. 2017;46:34-5.	Excluded by title
206	Sukurica Y, Gurel HG, Mutlu N. Six year follow-up of a patient treated with mandibular symphyseal distraction osteogenesis. Journal of Cranio-Maxillofacial Surgery. 2010;38(1):26-31.	Excluded by title
207	Suri L, Taneja P. Surgically assisted rapid palatal expansion: A literature review. Am J Orthod Dentofacial Orthop 2008;133(2):290-302.	Excluded by title
208	Suzuki SS, Previdente LH, Garcez AS, Suzuki H. Camouflage treatment of severe bialveolar protrusion in skeletal Class II using miniscrew anchorage. Int J Orthod Milwaukee. 2013;24(1):51-5.	Excluded by title
209	Swennen G, Dujardin T, Goris A, De Mey A, Malevez C. Maxillary distraction osteogenesis: a method with skeletal anchorage. J Craniofac Surg. 2000;11(2):120-7.	Excluded by title
210	Szuhanek C, Nagib R, Paraschivescu E, Cristina Bratu D, Brad S, Malita D. Digital facial and cephalometric analysis in the orthodontic treatment of a class III high angle patient: A case report. International Journal of Biology and Biomedical Engineering. 2015;9:109-13.	Excluded by title
211	Tausche E, Deeb W, Hansen L, Hietschold V, Harzer W, Schneider M. CT analysis of nasal volume changes after surgically-assisted rapid maxillary expansion. J Orofac Orthop. 2009;70(4):306-17.	Excluded by title
212	Thakkar S. Re: Dentoskeletal effects of a temporary skeletal anchorage device-supported rapid maxillary expansion appliance (TSADRME): A pilot study. Jason William Vassara, Anastasios Karydisb, Terry Trojan, Jack Fisher. The Angle Orthod 2016; 86: 241-249. Angle Orthod 2017;87(4):635-.	Excluded by title
213	Uribe F, Agarwal S, Janakiraman N, Shafer D, Nanda R. Bidimensional dentoalveolar distraction osteogenesis for treatment efficiency. Am J Orthod Dentofacial Orthop. 2013;144(2):290-8.	Excluded by title
214	Verlinden CR, Gooris PG, Becking AG. Complications in transpalatal distraction osteogenesis: a retrospective clinical study. J Oral Maxillofac Surg. 2011;69(3):899-905.	Excluded by title
215	Verquin M, Daems L, Politis C. Short-term complications after surgically assisted rapid palatal expansion: a retrospective cohort study. International Journal of Oral and Maxillofacial Surgery. 2017;46(3):303-8.	Excluded by title
216	Verstraaten J, Kuijpers-Jagtman AM, Mommaerts MY, Berge SJ, Nada RM, Schols JG. A systematic review of the effects of bone-borne surgical assisted rapid maxillary expansion. J Craniomaxillofac Surg. 2010;38(3):166-74.	Excluded by title
217	Vyas RM, Jarrahy R, Sisodia M, Jourabchi N, Wasson KL, Bradley JP. Bone-borne palatal distraction to correct the constricted cleft maxilla. J Craniofac Surg. 2009;20(3):733-6.	Excluded by title
218	Vyas RM, Jarrahy R, Sisodia M, Jourabchi N, Wasson KL, Bradley JP. Bone-Borne Palatal Distraction to Correct the Constricted Cleft Maxilla. Journal of Craniofacial Surgery. 2009;20(3):733-6.	Excluded by title
219	Walter A, Wendl B, Ploder O, Mojal S, Puigdollers A. Stability determinants of bone-borne force-transmitting components in three RME hybrid expanders-an in vitro study. Eur J Orthod 2017;39(1):76-84.	Excluded by title
220	Wang DZ, Chen G, Liao YM, Liu SG, Gao ZW, Hu J, et al. A new approach to repairing cleft palate and acquired palatal defects with distraction osteogenesis. International Journal of Oral and Maxillofacial Surgery. 2006;35(8):718-26.	Excluded by title
221	Wang JY, Zhang DL, Li MH. A three-dimensional positioning guide for accurate mini-screw insertion. Journal of Clinical Rehabilitative	Excluded by title

	Tissue Engineering Research. 2009;13(13):2593-6.	
222	Wang MH, Ge ZL, Tian L, Li PR, Che YQ. [Effect of three types of rapid maxillary expansion: a three-dimensional finite element study]. Zhonghua Kou Qiang Yi Xue Za Zhi. 2017;52(11):678-83.	Excluded by title
223	Wehrbein H, Gollner P. Skeletal anchorage in orthodontics basics and clinical application. Journal of Orofacial Orthopedics-Fortschritte Der Kieferorthopadie. 2007;68(6):443-61.	Excluded by title
224	Wilmes B, Su YY, Sadigh L, Drescher D. Pre-drilling force and insertion torques during orthodontic mini-implant insertion in relation to root contact. Journal of Orofacial Orthopedics. 2008;69(1):51-8.	Excluded by title
225	Xu HS, Ke K, Zhang ZL, Luo X, Jin X, Liu SSY, et al. Effects of Platelet-Rich Plasma and Recombinant Human Bone Morphogenetic Protein-2 on Suture Distraction Osteogenesis. Journal of Craniofacial Surgery. 2013;24(2):645-50.	Excluded by title
226	Yanagita T, Komori H, Tanaka T, Kamioka H. A case of adult cleft palate patient treatment with differential maxillary lateral expand distraction osteogenesis using combined expansion appliances. Orthodontic Waves. 2018;77(3):176-88.	Excluded by title
227	Yanagita T, Nakamura M, Kawanabe N, Yamashiro T. Class II malocclusion with complex problems treated with a novel combination of lingual orthodontic appliances and lingual arches. Am J Orthod Dentofacial Orthop 2014;146(1):98-107.	Excluded by title
228	Yáñez-Vico RM, Iglesias-Linares A, De Llano-Pérula MC, Solano-Reina A, Solano-Reina E. Management of occlusal canting with miniscrews. Angle Orthod 2014;84(4):737-47.	Excluded by title
229	Yatabe Ioshida MS. Three-dimensional assessment of maxillary protraction with miniplates in patients with cleft lip and palate. 2016:81-.	Excluded by title
230	Yatabe M, Garib D, Faco R, de Clerck H, Souki B, Janson G, et al. Mandibular and glenoid fossa changes after bone-anchored maxillary protraction therapy in patients with UCLP: A 3-D preliminary assessment. Angle Orthod 2017;87(3):423-31.	Excluded by title
231	Yatabe M, Garib DG, Faco RAS, de Clerck H, Janson G, Nguyen T, et al. Bone-anchored maxillary protraction therapy in patients with unilateral complete cleft lip and palate: 3-dimensional assessment of maxillary effects. Am J Orthod Dentofacial Orthop. 2017;152(3):327-35.	Excluded by title
232	Yilmaz HN, Garip H, Satilmis T, Kucukkeles N. Corticotomy-assisted maxillary protraction with skeletal anchorage and Class III elastics. Angle Orthod. 2015;85(1):48-57.	Excluded by title
233	Yurttadur G, Ileri Z, Akin M. Hybrid Rapid Palatal Expansion With 2 Mini-Screws: A Case Report. Turkish Journal of Orthodontics. 2015;28(2):64-70.	Excluded by title
234	Zandi M, Miresmaeili A, Heidari A. Short-term skeletal and dental changes following bone-borne versus tooth-borne surgically assisted rapid maxillary expansion: a randomized clinical trial study. J Craniomaxillofac Surg. 2014;42(7):1190-5.	Excluded by title
235	Zere E, Chaudhari PK, Sharan J, Dhingra K, Tiwari N. Developing Class III malocclusions: challenges and solutions. Clinical Cosmetic and Investigational Dentistry. 2018;10:99-116.	Excluded by title
236	Akay MC, Aras I, Günbay T, Aras A. Does transpalatal distraction affect pharyngeal airway dimensions and related soft tissues? Journal of Oral and Maxillofacial Surgery. 2014;72(8):1559-64.	Excluded by abstract
237	Akin M, Akgul YE, Ileri Z, Basciftci FA. Three-dimensional evaluation of hybrid expander appliances: A pilot study. Angle Orthodontist. 2016;86(1):81-6.	Excluded by abstract
238	Ali D, Mohammed H, Koo SH, Kang KH, Kim SC. Three-dimensional evaluation of tooth movement in Class II malocclusions treated without extraction by orthodontic mini-implant anchorage. Korean J Orthod 2016;46(5):280-9.	Excluded by abstract
239	Al-Mozany SA, Dalci O, Almuzian M, Gonzalez C, Tarraf NE, Ali Darendeliler M. A novel method for treatment of Class III malocclusion in growing patients. Prog Orthod. 2017;18(1):40.	Excluded by abstract
240	Amini F, Poosti M. A new approach to correct a Class III malocclusion with miniscrews: a case report. Journal of the California Dental Association. 2013;41(3):197-200.	Excluded by abstract
241	Aras A, Akay MC, Cukurova I, Gunbay T, Isiksal E, Aras I. Dimensional Changes of the Nasal Cavity After Transpalatal Distraction Using Bone-Borne Distractor: An Acoustic Rhinometry and Computed Tomography Evaluation. Journal of Oral and Maxillofacial Surgery. 2010;68(7):1487-97.	Excluded by abstract
242	Asan CY, Kutuk N, Kurt G, Alkan A. Development of a new three-directional distractor system for the correction of maxillary transverse and sagittal deficiency. Journal of Cranio-Maxillofacial Surgery. 2018;46(3):424-31.	Excluded by abstract
243	Buschang PH, Carrillo R, Rossouw PE. Orthopedic correction of growing hyperdivergent, retrognathic patients with miniscrew implants. Journal of Oral and Maxillofacial Surgery. 2011;69(3):754-62.	Excluded by abstract
244	Caminiti MF, Marko JV, Metaxas A. TAD retained acrylic palatal expander for SARPE. Journal of Oral and Maxillofacial Surgery. 2017;75(10):e349-e50.	Excluded by abstract
245	Carneiro Jr JT, Paschoal EHA, Carreira ASD, Real RPV. Carotid cavernous fistula after surgically assisted rapid maxillary expansion with a bone anchored appliance. International Journal of Oral and Maxillofacial Surgery. 2013;42(3):326-8.	Excluded by abstract
246	Charezinski M, Balon-Perin A, Deroux E, De Maertelaer V, Glineur R. Transverse maxillary stability assisted by a transpalatal device: a retrospective pilot study of 9 cases. Int J Oral Maxillofac Surg. 2009;38(9):937-41.	Excluded by abstract
247	Chen Y, Kim KA, Seo KW, Kang YG, Oh SH, Choi YS, et al. A New Designed Expander Supported by Spike Miniscrews With Enhanced Stability. J Craniofac Surg. 2016;27(2):e130-3.	Excluded by abstract
248	Choi SH, Cha JY, Kang DY, Hwang CJ. Surgical-orthodontic treatment for skeletal class II malocclusion with vertical maxillary excess, anterior open bite, and transverse maxillary deficiency. J Craniofac Surg. 2012;23(6):e531-5.	Excluded by abstract
249	Cortese A, Savastano M, Savastano G, Claudio PP. One-step transversal palatal distraction and maxillary repositioning: technical considerations, advantages, and long-term stability. J Craniofac Surg. 2011;22(5):1714-9.	Excluded by abstract
250	Cortese A, Savastano M, Savastano G, Claudio PP. One-Step Transversal Palatal Distraction and Maxillary Repositioning: Technical Considerations, Advantages, and Long-Term Stability. Journal of Craniofacial Surgery. 2011;22(5):1714-9.	Excluded by abstract
251	De Clerck HJ, Proffit WR. Growth modification of the face: A current perspective with emphasis on Class III treatment. Am J Orthod Dentofacial Orthop 2015;148(1):37-46.	Excluded by abstract
252	De Gijt P, Van Der Wal KG, Wolvius EB, Koudstaal MJ. Eight years of bimaxillary transverse expansion. International Journal of Oral and Maxillofacial Surgery. 2011;40(10):1037.	Excluded by abstract
253	Dobrzyński M, Miśków K, Dowgierd K. Interception of developing class III malocclusion with temporary anchorage devices: A case report. Polish Annals of Medicine. 2014;21(2):131-5.	Excluded by abstract
254	Fedder C, Hengster V, Kuehn J, Kretschmer W. Comparison of symmetry of rapid maxillary expansion using a tooth-borne or a bone-borne distractor. International Journal of Oral and Maxillofacial Surgery. 2017;46:156.	Excluded by abstract
255	Fernandez-Sanroman J, Donascimento MG, Lopez AC, Ferro MF, Berrondo IA. Transverse maxillary distraction in patients with periodontal pathology or insufficient tooth anchorage using custom-made devices. J Oral Maxillofac Surg. 2010;68(7):1530-6.	Excluded by abstract
256	Gerlach KL, Zahl C. Transversal palatal expansion using a palatal distractor. J Orofac Orthop. 2003;64(6):443-9.	Excluded by abstract
257	González de Bello M, André Lisbôa SH, Cotrim-Ferreira FA, Rívea IF. Tratamiento no quirúrgico de las mordidas abierta esqueletales: utilización adecuada del anclaje con mini-implantes. Acta Odontol Venez. 2012;50(1).	Excluded by abstract
258	Hansen L, Tausche E, Hietschold V, Hotan T, Lagravere M, Harzer W. Skeletally-anchored rapid maxillary expansion using the Dresden Distractor. J Orofac Orthop. 2007;68(2):148-58.	Excluded by abstract
259	Hernandez-Alfaro F, Bueno JM, Diaz A, Pagés CM. Minimally invasive surgically assisted rapid palatal expansion with limited approach under sedation: A report of 283 consecutive cases. Journal of Oral and Maxillofacial Surgery. 2010;68(9):2154-8.	Excluded by abstract
260	Iida S, Haraguchi S, Aikawa T, Yashiro K, Okura M, Kogo M. Conventional bone-anchored palatal distractor using an orthodontic palatal expander for the transverse maxillary distraction osteogenesis: technical note. Oral Surgery, Oral Medicine, Oral Pathology, Oral Radiology and Endodontology. 2008;105(2):e8-e11.	Excluded by abstract
261	Jamilian A, Showkatbakhsh R. Treatment of maxillary deficiency by miniscrew implants--a case report. J Orthod. 2010;37(1):56-61.	Excluded by abstract
262	Kale B, Buyukcavus MH, Esenlik E. Comparison of the change in inferior sclera exposure after maxillary protraction with or without skeletal anchorage. Niger J Clin Pract. 2018;21(7):854-8.	Excluded by abstract
263	Kim KY, Bayome M, Park JH, Kim KB, Mo SS, Kook YA. Displacement and stress distribution of the maxillofacial complex during maxillary protraction with buccal versus palatal plates: finite element analysis. Eur J Orthod 2015;37(3):275-83.	Excluded by abstract
264	Kim SY, Park YC, Lee KJ, Lintermann A, Han SS, Yu HS, et al. Assessment of changes in the nasal airway after nonsurgical miniscrew-assisted rapid maxillary expansion in young adults. Angle Orthodontist. 2018;88(4):435-41.	Excluded by abstract
265	Kobayashi M, Fushima K. Orthodontic skeletal anchorage using a palatal external plate. Journal of Orthodontics. 2014;41(1):53-62.	Excluded by abstract

266	Kolk A, Bissinger O, Wolff KD, Jüngling K, Winsauer H, Ploder O. The maxillary expansion with different devices. International Journal of Oral and Maxillofacial Surgery. 2015;44:e93.	Excluded by abstract
267	Koudstaal MJ, van der Wal KG, Wolvius EB, Schulten AJ. The Rotterdam Palatal Distractor: introduction of the new bone-borne device and report of the pilot study. Int J Oral Maxillofac Surg. 2006;35(1):31-5.	Excluded by abstract
268	Koudstaal MJ, van der Wal KGH, Wolvius EB, Schulten AJM. The Rotterdam Palatal Distractor: Introduction of the new bone-borne device and report of the pilot study. International Journal of Oral and Maxillofacial Surgery. 2006;35(1):31-5.	Excluded by abstract
269	Kwon HY, Mah SJ, Kang YG. Asymmetric transverse control of maxillary dentition with two midpalatal orthodontic miniscrews. Angle Orthodontist. 2015;85(3):525-34.	Excluded by abstract
270	Laino L, Troiano G, Dioguardi M, Perillo L, Laino G, Lo Muzio L, et al. Patient Discomfort During and After Surgically Assisted Rapid Maxillary Expansion Under Local Anaesthesia. Journal of Craniofacial Surgery. 2016;27(3):772-5.	Excluded by abstract
271	Landes CA, Laudemann K, Petruchin O, Mack MG, Kopp S, Ludwig B, et al. Comparison of Bipartite Versus Tripartite Osteotomy for Maxillary Transversal Expansion Using 3-Dimensional Preoperative and Postexpansion Computed Tomography Data. Journal of Oral and Maxillofacial Surgery. 2009;67(10):2287-301.	Excluded by abstract
272	Laudemann K, Petruchin O, Mack MG, Kopp S, Sader R, Landes CA. Evaluation of surgically assisted rapid maxillary expansion with or without pterygomaxillary disjunction based upon preoperative and post-expansion 3D computed tomography data. Oral and Maxillofacial Surgery. 2009;13(3):159-69.	Excluded by abstract
273	Laudemann K, Santo G, Revilla C, Harth M, Kopp S, Sader RA, et al. Assessment of surgically assisted rapid maxillary expansion regarding pterygomaxillary disjunction using thin volume-rendering technique: In variance analysis and in reliability, accuracy, and validity. Journal of Oral and Maxillofacial Surgery. 2011;69(10):2631-43.	Excluded by abstract
274	Lee HK, Bayome M, Ahn CS, Kim SH, Kim KB, Mo SS, et al. Stress distribution and displacement by different bone-borne palatal expanders with micro-implants: a three-dimensional finite-element analysis. Eur J Orthod. 2014;36(5):531-40.	Excluded by abstract
275	Lee JH, Choo H, Kim SH, Chung KR, Giannuzzi LA, Ngan P. Replacing a failed mini-implant with a miniplate to prevent interruption during orthodontic treatment. American Journal of Orthodontics and Dentofacial Orthopedics. 2011;139(6):849-57.	Excluded by abstract
276	Lee NK, Yang IH, Baek SH. The short-term treatment effects of face mask therapy in Class III patients based on the anchorage device Miniplates vs rapid maxillary expansion. Angle Orthod 2012;82(5):846-52.	Excluded by abstract
277	Lee RJ, Moon W, Hong C. Effects of monocortical and bicortical mini-implant anchorage on bone-borne palatal expansion using finite element analysis. Am J Orthod Dentofacial Orthop. 2017;151(5):887-97.	Excluded by abstract
278	Lee SC, Park JH, Bayome M, Kim KB, Araujo EA, Kook YA. Effect of bone-borne rapid maxillary expanders with and without surgical assistance on the craniofacial structures using finite element analysis. Am J Orthod Dentofacial Orthop. 2014;145(5):638-48.	Excluded by abstract
279	Leyder P, Altounian G, Chardain J, Quilichini J. Adjustable selective maxillary expansion combined with maxillomandibular surgery: A case report. Int Orthod. 2015;13(3):320-31.	Excluded by abstract
280	Lima ANd, Detoni E, Milani BdA, Morando FS, Jorge WA. Dispositivo ósseo-suportado para expansão maxilar: relatos de casos. Rev cir traumatol buco-maxilo-fac. 2011;11(4):19-24.	Excluded by abstract
281	Mathew A, Nagachandran KS, Vijayalakshmi D. Stress and displacement pattern evaluation using two different palatal expanders in unilateral cleft lip and palate: a three-dimensional finite element analysis. Prog Orthod. 2016;17(1):38.	Excluded by abstract
Exclusion by full text		
282	Machado FRC, Dums K, Losso EM, Correr GM, Moro A, Moresca R. Alterações dentárias transversais em pacientes submetidos à expansão rápida da maxila com ancoragem esquelética. Ortho Sci, Orthod sci pract. 2013;6(23):291-9.	Excluded; missing fulltext
283	Matteini C, Mommaerts MY. Posterior transpalatal distraction with pterygoid disjunction: a short-term model study. Am J Orthod Dentofacial Orthop. 2001;120(5):498-502.	Excluded by abstract
284	Mommaerts MY. Transpalatal distraction as a method of maxillary expansion. British Journal of Oral and Maxillofacial Surgery. 1999;37(4):268-72.	Excluded by abstract
285	Moon W, Wu KW, MacGinnis M, Sung J, Chu H, Youssef G, et al. The efficacy of maxillary protraction protocols with the micro-implant-assisted rapid palatal expander (MARPE) and the novel N2 mini-implant-a finite element study. Prog Orthod. 2015;16:16.	Excluded by abstract
286	Nada RM, Fudalej PS, Maal TJJ, Berge SJ, Mostafa YA, Kuijpers-Jagtman AM. Three-dimensional prospective evaluation of tooth-borne and bone-borne surgically assisted rapid maxillary expansion. Journal of Cranio-Maxillofacial Surgery. 2012;40(8):757-62.	Excluded by abstract
287	Nada RM, van Loon B, Maal TJJ, Berge SJ, Mostafa YA, Kuijpers-Jagtman AM, et al. Three-dimensional evaluation of soft tissue changes in the orofacial region after tooth-borne and bone-borne surgically assisted rapid maxillary expansion. Clinical Oral Investigations. 2013;17(9):2017-24.	Excluded by abstract
288	Nada RM, van Loon B, Schols J, Maal TJJ, de Koning MJ, Mostafa YA, et al. Volumetric changes of the nose and nasal airway 2 years after tooth-borne and bone-borne surgically assisted rapid maxillary expansion. European Journal of Oral Sciences. 2013;121(5):450-6.	Excluded by abstract
289	Nalabothu P, Syed AH, Chitharanjan A, Murthy J. The stability of combined maxillary and transpalatal distraction in patients with cleft lip and palate. Cleft Palate-Craniofacial Journal. 2014;51(3):e56.	Excluded by abstract
290	Nelson Elias C, Jogaib Fernandes D, Souza Zanivan D, Resende Fonseca Y. Extensiometric analysis of strain in craniofacial bones during implant-supported palatal expansion. Journal of the Mechanical Behavior of Biomedical Materials. 2017;76:104-9.	Excluded by abstract
291	Nevzatoglu S, Kucukkeles N, Guzel Z. Long-term stability of intra-oral maxillary distraction in unilateral cleft lip and palate: a case report. Aust Orthod J. 2013;29(2):200-8.	Excluded by abstract
292	Neyt NMF, Mommaerts MY, Abeloos JVS, De Clercq CAS, Neyt LF. Problems, obstacles and complications with transpalatal distraction in non-congenital deformities. Journal of Cranio-Maxillofacial Surgery. 2002;30(3):139-43.	Excluded by abstract
293	Ngan P, Moon W. Evolution of Class III treatment in orthodontics. Am J Orthod Dentofacial Orthop 2015;148(1):22-36.	Excluded by abstract
294	Nguyen T, Cevidanes L, Paniagua B, Zhu HT, Koerich L, De Clerck H. Use of shape correspondence analysis to quantify skeletal changes associated with bone-anchored Class III correction. Angle Orthod 2014;84(2):329-36.	Excluded by abstract
295	Nikolaev AV, Andreishchev AR, Kutukova SI. [Comparative biomechanical study of surgically assisted rapid palatal expansion with tooth-borne and bone-borne expanders]. Stomatologiia (Mosk). 2017;96(5):48-55.	Excluded by abstract
296	Oliveira NKd, Nascimento FFAdO, Chaves Netto HDdM, Mazzonetto R. Distrator transpalatino em expansão cirúrgica de maxila: considerações atuais e relato de caso. Ortho Sci, Orthod sci pract. 2009;2(7/8):765-9.	Excluded by abstract
297	Pan HH, Huang Y, Yang Z, Yang SW. [3-D finite element study on rapid maxillary expansion using implant anchorage]. Sichuan Da Xue Xue Bao Yi Xue Ban. 2011;42(6):831-4.	Excluded by abstract
298	Parel SM, Branemark PI, Ohrnell LO, Svensson B. Remote implant anchorage for the rehabilitation of maxillary defects. J Prosthet Dent. 2001;86(4):377-81.	Excluded by abstract
299	Parel SM, Brånemark PI, Ohrnell LO, Svensson B. Remote implant anchorage for the rehabilitation of maxillary defects. Journal of Prosthetic Dentistry. 2001;86(4):377-81.	Excluded by abstract
300	Parhiz A, Schepers S, Lambrichts I, Vrielinck L, Sun Y, Politis C. Lateral cephalometry changes after SARPE. International Journal of Oral and Maxillofacial Surgery. 2011;40(7):662-71.	Excluded by abstract
301	Pinto PX, Mommaerts MY, Wreakes G, Jacobs WV. Immediate postexpansion changes following the use of the transpalatal distractor. J Oral Maxillofac Surg. 2001;59(9):994-1000; discussion 1.	Excluded by abstract
302	Pinto PX, Pinto PX, Mommaerts MY, Wreakes G, Jacobs WVGJA. Immediate postexpansion changes following the use of the transpalatal distractor. Journal of Oral and Maxillofacial Surgery. 2001;59(9):994-1000.	Excluded by abstract
303	Pithon MM. Nonsurgical treatment of severe Class II malocclusion with anterior open bite using mini-implants and maxillary lateral incisor and mandibular first molar extractions. Am J Orthod Dentofacial Orthop 2017;151(5):964-77.	Excluded by abstract
304	Ramieri GA, Spada MC, Austa M, Bianchi SD, Berrone S. Transverse maxillary distraction with a bone-anchored appliance: dento-periodontal effects and clinical and radiological results. Int J Oral Maxillofac Surg. 2005;34(4):357-63.	Excluded by abstract
305	Reyes Doimi J, Aguirre Balseca GM, Cáceres La Torre A. Colocación de implantes dentales en maxilar atrófico con técnica de cresta dividida y expansión del reborde. Rev odontol mex. 2017;21(3):198-204.	Excluded by abstract
306	Santos SE, Gonçalves GM, Loureiro FR, Lopes MCdA, Moreira RWF. Distrator palatal de Rotterdam: uma opção para expansão cirúrgica de maxila. Rev cir traumatol buco-maxilo-fac. 2012;12(4):21-6.	Excluded by abstract
307	Sar C, Sahinoglu Z, Ozcirpici AA, Uckan S. Dentofacial effects of skeletal anchored treatment modalities for the correction of maxillary retrognathia. Am J Orthod Dentofacial Orthop. 2014;145(1):41-54.	Excluded by abstract

308	Scolozzi P, Verdeja R, Herzog G, Jaques B. Maxillary expansion using transpalatal distraction in patients with unilateral cleft lip and palate. Plast Reconstr Surg. 2007;119(7):2200-5.	Excluded by abstract
309	Seeberger R, Abe-Nickler D, Hoffmann J, Kunzmann K, Zingler S. One-stage tooth-borne distraction versus two stage bone-borne distraction in surgically assisted maxillary expansion (SARME). Oral Surgery, Oral Medicine, Oral Pathology and Oral Radiology. 2015;120(6):693-8.	Excluded by abstract
310	Seitz O, Landes C, Dissmann JP, Sader R, Klein C. [First experiences in maxillary arch expansion using the MWD (Maxillary Widening Device)]. Mund Kiefer Gesichtschir. 2007;11(6):327-32.	Excluded by abstract
311	Seitz O, Landes C, Dissmann JP, Sader R, Klein C. First experiences in maxillary arch expansion using the MWD™. Mund - Kiefer - und Gesichtschirurgie. 2007;11(6):327-32.	Excluded by abstract
312	Seo YJ, Chung KR, Kim SH, Nelson G. Camouflage treatment of skeletal Class III malocclusion with asymmetry using a bone-borne rapid maxillary expander. Angle Orthodontist. 2015;85(2):322-34.	Excluded by abstract
313	Seo YJ, Lin L, Kim SH, Chung KRP, Nelson GP. Strategic camouflage treatment of skeletal Class III malocclusion (mandibular prognathism) using bone-borne rapid maxillary expansion and mandibular anterior subapical osteotomy. American Journal of Orthodontics and Dentofacial Orthopedics. 2016;149(1):114-26.	Excluded by abstract
314	Swennen GR, Treutlein C, Brachvogel P, Berten JL, Schwestka-Polly R, Hausamen JE. Segmental unilateral transpalatal distraction in cleft patients. J Craniofac Surg. 2003;14(5):786-90.	Excluded by abstract
315	Tausche E, Deeb W, Hansen L, Hietschold V, Harzer W, Schneider M. CT analysis of nasal volume changes after surgically-assisted rapid maxillary expansion. Journal of Orofacial Orthopedics. 2009;70(4):306-17.	Excluded by abstract
316	Uribe F, Agarwal S, Janakiraman N, Shafer D, Nanda R. Bidimensional dentoalveolar distraction osteogenesis for treatment efficiency. American Journal of Orthodontics and Dentofacial Orthopedics. 2013;144(2):290-8.	Excluded by abstract
317	Wendl B, Winsauer H, Walter A, Muchitsch AP, Jakse N. Borderline Treatment in Patients with Transversal Deficits. Informationen Aus Orthodontie Und Kieferorthopaedie. 2017;49(4):262-70.	Excluded by abstract
318	Wilmes B, Ludwig B, Katyal V, Nienkemper M, Rein A, Drescher D. The Hybrid Hyrax Distalizer, a new all-in-one appliance for rapid palatal expansion, early class III treatment and upper molar distalization. J Orthod. 2014;41 Suppl 1:S47-53.	Excluded by abstract
319	Wilmes B, Ludwig B, Katyal V, Nienkemper M, Rein A, Drescher D. The Hybrid Hyrax distalizer, a new all-in-one appliance for rapid palatal expansion, early class III treatment and upper molar distalization. Journal of Orthodontics. 2014;41:S47-S53.	Excluded by abstract
320	Yan XL, He WJ, Lin T, Liu J, Bai XF, Yan GQ, et al. Three-dimensional finite element analysis of the craniomaxillary complex during maxillary protraction with bone anchorage vs conventional dental anchorage. Am J Orthod Dentofacial Orthop 2013;143(2):197-205.	Excluded by abstract
321	Yılmaz Asan C, Kütük N, Kurt G, Alkan A. Development of a new three-directional distractor system for the correction of maxillary transverse and sagittal deficiency. Journal of Cranio-Maxillofacial Surgery. 2018;46(3):398-412.	Excluded by abstract
322	Zhang XJ, Zheng CS, Zhang M. [Treatment of Class II malocclusion using Herbst appliance with headgear and rapid palatal expansion and straight wire appliance with implant anchorage]. Shanghai Kou Qiang Yi Xue. 2015;24(3):361-6.	Excluded by abstract
323	{NCT01164631}. Pharyngeal Size in Patients With Obstructive Tonsils Under Orthodontic Treatment. Completed	Excluded; ongoing
324	{NCT01765062}. One Year Skeletal and Dental Arch Changes Following Rapid Maxillary Expansion Treatment in Children. Completed	Excluded; ongoing
325	{NCT01837914}. Maxillary Expansion Treatment of Pediatric OSA. Recruiting	Excluded; ongoing
326	{NCT02178826}. Nocturnal Enuresis and Rapid Maxillary Expansion. Recruiting	Excluded; ongoing
327	{NCT02424422}. The Effect of Expansion on Craniofacial Sutures in Children Using 3D Imaging. Completed	Excluded; ongoing
328	{NCT02798822}. Maxillary and Mandibular Arch Response to RME: a Multicentric Randomized Controlled Trial. Completed	Excluded; ongoing
329	{NCT02810353}. Analysis of the Dentoskeletal Effects of the Expander With Differential Opening. Recruiting	Excluded; ongoing
330	{NCT02947464}. Rapid Maxillary Expansión for Residual Pediatric Obstructive Sleep Apnea After Adenotonsillectomy. Recruiting	Excluded; ongoing
331	{NCT03004300}. Maxillary Expansion Effects in Children With Upper Airway Obstruction. Recruiting	Excluded; ongoing
332	{NCT03028038}. Using Platelet Rich Plasma in Reducing Alveolar Bone Resorption During Rapid Maxillary Expansion. Active, not recruiting	Excluded; ongoing
333	{NCT03159962}. Mandibular Response After Maxillary Orthopedic Expansion in Class II Growing Subjects. Completed	Excluded; ongoing
334	{NCT03353766}. Early Crossbite Correction - a Randomized Study. Recruiting	Excluded; ongoing
335	Chane-Fane C, Darqué F. Rapid maxillary expansion assisted by palatal mini-implants in adolescents - Preliminary study. International Orthodontics. 2015;13(1):96-111.	Excluded; missing fulltext
336	Ellouze S, Darque F. Mini-implant-assisted expansion. Mini-Implants: Orthodontics of the Future2014. p. 217-28.	Excluded; missing fulltext
337	Farhangfar A, Bogowicz P, Heo G, Lagravere MO. Palatal bone resorption in bone-anchored maxillary expander treatment. Int Orthod. 2012;10(3):274-88.	Excluded; missing fulltext
338	García Baeza J, Bejarano E, Secchi AG, Hajibaik N, Vanarsdall RL. Comparación de la expansión maxilar en gemelos idénticos obtenida con dos tipos de disyuntor palatino: anclaje esquelético y anclaje dentario. Ortod esp (Ed impr). 2011;51(1):50-8.	Excluded; missing fulltext
339	Pham V, Lagravere MO. Alveolar bone level changes in maxillary expansion treatments assessed through CBCT. Int Orthod. 2017;15(1):103-13.	Excluded; missing fulltext
340	Poncio AMM. Tratamento da má-oclusão de classe III com ancoragem esquelética. Ortodontia. 2016;49(1):61-5.	Excluded; missing fulltext
341	Ribeiro JRL, Moresca R, Gomes R, Moro A, Guarita MK, Correr GM. Intercorrências durante a ERM com ancoragem esquelética. Ortho Sci, Orthod sci pract. 2013;6(22):170-7.	Excluded; missing fulltext
342	Tausche E, Hansen L, Schneider M, Harzer W. [Bone-supported rapid maxillary expansion with an implant-borne Hyrax screw: the Dresden Distractor]. Orthod Fr. 2008;79(2):127-35.	Excluded; missing fulltext
343	Wang B, Fang B, Xia YH, Mao LX. [Exploration on the sliding mechanism of implant anchorage assisted self-ligating appliance]. Shanghai Kou Qiang Yi Xue. 2010;19(5):449-55.	Excluded; missing fulltext
344	Koudstaal MJ, van der Wal KGH, Wolvius EB. The Rotterdam palatal distractor. Proceedings of the 5th International Congress of Maxillofacial and Craniofacial Distraction2006. p. 167-+.	Excluded; missing fulltext
345	Morales-Fernandez M, Iglesias-Linares A, Yanez-Vico RM, Mendoza-Mendoza A, Solano-Reina E. Bone- and dentoalveolar-anchored dentofacial orthopedics for Class III malocclusion: New approaches, similar objectives? A systematic review. Angle Orthod 2013;83(3):540-52.	Excluded; not relevant
346	Sahin T, Delforge A, Garreau E, Raoul G, Ferri J. Orthopedic treatment of Class III malocclusions using skeletal anchorage: A bibliographical review. International Orthodontics. 2016;14(3):263-72.	Excluded; not relevant
347	Wilmes B, Olthoff G, Drescher D. Comparison of skeletal and conventional anchorage methods in conjunction with pre-operative decompensation of a skeletal class III malocclusion. J Orofac Orthop. 2009;70(4):297-305.	Excluded; not relevant
348	Leyder P, Altounian G, Quilichini J. Adjustable selective maxillary expansion combined with one-stage maxillomandibular surgery: A prospective study of osseous widening in fifty-five consecutive patients. Journal of Cranio-Maxillofacial Surgery. 2018;46(9):1408-20.	Excluded; not relevant
349	Gandedkar NH, Koo CS, Sharan J, Chng CK, Vaid N. The temporary anchorage devices research terrain: Current perspectives and future forecasts! Seminars in Orthodontics. 2018;24(1):191-206.	Excluded; no clinical study
350	Garib DG, Navarro RdL, Francischone CE, Oltramari PVP. Expansão rápida da maxila ancorada em implantes: uma nova proposta para expansão ortopédica na dentadura permanente. Rev Dent Press Ortod Ortop Facial (Impr). 2007;12(3):75-81.	Excluded; no clinical study
351	Lee KJ, Choi SH, Choi TH, Shi KK, Keum BT. Maxillary transverse expansion in adults: Rationale, appliance design, and treatment outcomes. Seminars in Orthodontics. 2018;24(1):52-65.	Excluded; no clinical study
352	Nowak RM, Strzałkowska A, Zawiślak E. Treatment options and limitations in transverse maxillary deficiency. Dental and Medical Problems. 2015;52(4):389-400.	Excluded; no clinical study
353	Roveta AL, Bolasco L. Disyunción con diferentes elementos de anclaje esqueletal. Actas odontol. 2013;10(2):19-27.	Excluded; no clinical study
354	Solano-Mendoza B, Iglesias-Linares A, Yanez-Vico RM, Mendoza-Mendoza A, Alio-Sanz JJ, Solano-Reina E. Maxillary Protraction at Early Ages. The Revolution of New Bone Anchorage Appliances. Journal of Clinical Pediatric Dentistry. 2012;37(2):219-29.	Excluded; no clinical study
355	Suzuki H, Moon W, Previdente LH, Suzuki SS, Garcez AS, Consolaro A. Miniscrew-assisted rapid palatal expander (MARPE): the quest for pure orthopedic movement. Dental Press J Orthod. 2016;21(4):17-23.	Excluded; no clinical study
356	Buck LM, Dalci O, Darendeliler MA, Papadopoulou AK. Effect of Surgically Assisted Rapid Maxillary Expansion on Upper Airway Volume: A Systematic Review. Journal of Oral and Maxillofacial Surgery. 2016;74(5):1025-43.	Excluded; systematic review
357	Verstraaten J, Kuijpers-Jagtman AM, Mommaerts MY, Bergé SJ, Nada RM, Schols JGJH. A systematic review of the effects of bone-borne surgical assisted rapid maxillary expansion. Journal of Cranio-Maxillofacial Surgery. 2010;38(3):166-74.	Excluded; systematic review

358	Algharbi M, Bazargani F, Dimberg L. Do different maxillary expansion appliances influence the outcomes of the treatment? European Journal of Orthodontics. 2018;40(1):97-106.	Excluded; systematic review
359	Tang Z, Jiang LP, Wu JY. Effect of maxillary expansion on orthodontics. Asian Pacific Journal of Tropical Medicine. 2015;8(11):922-8.	Excluded; animal study
360	Adolphs N, Ernst N, Menneking H, Hoffmeister B. Transpalatal distraction - State of the art for the individual management of transverse maxillary deficiency - A review of 50 consecutive cases. Journal of Cranio-Maxillofacial Surgery. 2014;42(8):1669-74.	Excluded; no conventional maxillary expansion
361	Aras I, Olmez S, Akay MC, Gunbay T, Aras A. The Effects of Maxillary Expansion on the Soft Tissue Facial Profile. Journal of Istanbul University Faculty of Dentistry. 2017;51(3):1-10.	Excluded; no conventional maxillary expansion
362	Aziz SR, Tanchyk A. Surgically Assisted Palatal Expansion With a Bone-Borne Self-Retaining Palatal Expander. Journal of Oral and Maxillofacial Surgery. 2008;66(9):1788-93.	Excluded; no conventional maxillary expansion
363	Cortese A, Savastano G, Amato M, Cantone A, Boschetti C, Claudio PP. New palatal distraction device by both bone-borne and tooth-borne force application in a paramedian bone anchorage site: surgical and occlusal considerations on clinical cases. J Craniofac Surg. 2014;25(2):589-95.	Excluded; no conventional maxillary expansion
364	Dowgierd K, Smektała T, Ulański Ł, Dowgierd M, Kozakiewicz M. Changes in transverse tooth and bone dimensions during transversal maxillary hypoplasia treatment by maxillary distraction osteogenesis using a device installed on a bone. Polish Annals of Medicine. 2015;22(2):110-8.	Excluded; no conventional maxillary expansion
365	Dowgierd K. Transverse maxillary distraction - Preliminary reports. International Journal of Oral and Maxillofacial Surgery. 2011;40(10):1193.	Excluded; no conventional maxillary expansion
366	Feng X, Yang Z, Dai J, Li R. Miniscrew anchorage for the correction of midline discrepancies. J Clin Orthod. 2014;48(3):169-79.	Excluded; no conventional maxillary expansion
367	Fernández-Sanromán J, Donascimento MG, López AC, Ferro MF, Berrondo IA. Transverse Maxillary Distraction in Patients With Periodontal Pathology or Insufficient Tooth Anchorage Using Custom-Made Devices. Journal of Oral and Maxillofacial Surgery. 2010;68(7):1530-6.	Excluded; no conventional maxillary expansion
368	Garreau E, Bouscaillou J, Rattier S, Ferri J, Raoul G. Bone-borne distractor versus tooth-borne distractor for orthodontic distraction after surgical maxillary expansion: The patient's point of view. International Orthodontics. 2016;14(2):214-32.	Excluded; no conventional maxillary expansion
369	Gunaseelan R, Cheung LK, Krishnaswamy R, Veerababu M. Anterior maxillary distraction by tooth-borne palatal distractor. Journal of Oral and Maxillofacial Surgery. 2007;65(5):1044-9.	Excluded; no conventional maxillary expansion
370	Gunbay T, Akay MC, Gunbay S, Aras A, Koyuncu BO, Sezer B. Transpalatal distraction using bone-borne distractor: clinical observations and dental and skeletal changes. J Oral Maxillofac Surg. 2008;66(12):2503-14.	Excluded; no conventional maxillary expansion
371	Gunbay T, Akay MC, Gunbay S, Aras A, Koyuncu BO, Sezer B. Transpalatal Distraction Using Bone-Borne Distractor: Clinical Observations and Dental and Skeletal Changes. Journal of Oral and Maxillofacial Surgery. 2008;66(12):2503-14.	Excluded; no conventional maxillary expansion
372	Hansen L, Tausche E, Hietschold V, Hotan T, Lagravère M, Harzer W. Skeletally-anchored rapid maxillary expansion using the dresden distractor. Journal of Orofacial Orthopedics. 2007;68(2):148-58.	Excluded; no conventional maxillary expansion
373	Harzer W, Reusser L, Hansen L, Richter R, Nagel T, Tausche E. Minimally invasive rapid palatal expansion with an implant-supported hyrax screw. Biomed Tech (Berl). 2010;55(1):39-45.	Excluded; no conventional maxillary expansion
374	Kang YG, Kim JY, Nam JH. Control of maxillary dentition with 2 midpalatal orthodontic miniscrews. Am J Orthod Dentofacial Orthop. 2011;140(6):879-85.	Excluded; no conventional maxillary expansion
375	Koudstaal MJ, van der Wal KG, Wolvius EB. Experience with the transpalatal distractor in congenital deformities. Mund Kiefer Gesichtschir. 2006;10(5):331-4.	Excluded; no conventional maxillary expansion
376	Koudstaal MJ, van der Wal KGH, Wolvius EB. The Rotterdam palatal distractor. Proceedings of the 5th International Congress of Maxillofacial and Craniofacial Distraction2006. p. 167-72.	Excluded; no conventional maxillary expansion
377	Koudstaal MJ, Wolvius EB, Schulten AJM, Hop WCJ, van der Wal KGH. Stability, tipping and relapse of bone-borne versus tooth-borne surgically assisted rapid maxillary expansion; a prospective randomized patient trial. International Journal of Oral and Maxillofacial Surgery. 2009;38(4):308-15.	Excluded; no conventional maxillary expansion
378	Kunz F, Linz C, Baunach G, Böhm H, Meyer-Marcotty P. Expansion patterns in surgically assisted rapid maxillary expansion: Transpalatal distractor versus hyrax appliance. Journal of Orofacial Orthopedics. 2016;77(5):357-65.	Excluded; no conventional maxillary expansion
379	Landes CA, Laudemann K, Schübel F, Petruchin O, MacK M, Kopp S, et al. Comparison of tooth- and bone-borne devices in surgically assisted rapid maxillary expansion by three-dimensional computed tomography monitoring: Transverse dental and skeletal maxillary expansion, segmental inclination, dental tipping, and vestibular bone resorption. Journal of Craniofacial Surgery. 2009;20(4):1132-41.	Excluded; no conventional maxillary expansion
380	Laudemann K, Petruchin O, Nafzger M, Ballon A, Kopp S, Sader RA, et al. Long-term 3D cast model study: Bone-borne vs. tooth-borne surgically assisted rapid maxillary expansion due to secondary variables. Oral and Maxillofacial Surgery. 2010;14(2):105-14.	Excluded; no conventional maxillary expansion
381	Malkoc S, Usumez S, Iseri H. Long-term effects of symphyseal distraction and rapid maxillary expansion on pharyngeal airway dimensions, tongue, and hyoid position. Am J Orthod Dentofacial Orthop. 2007;132(6):769-75.	Excluded; no conventional maxillary expansion
382	Mommaerts MY. Transpalatal distraction as a method of maxillary expansion. Br J Oral Maxillofac Surg. 1999;37(4):268-72.	Excluded; no conventional maxillary expansion
383	Ramieri GA, Nasi A, Dell'acqua A, Verze L. Facial soft tissue changes after transverse palatal distraction in adult patients. Int J Oral Maxillofac Surg. 2008;37(9):810-8.	Excluded; no conventional maxillary expansion
384	Sari E, Kadioglu O, Ucar C, Altug HA. Prostaglandin E2 levels in gingival crevicular fluid during tooth- and bone-borne expansion. Eur J Orthod. 2010;32(3):336-41.	Excluded; no conventional maxillary expansion
385	Seitz O, Landes CA, Philipp DJ, Sader R, Klein CM. Reliable surgically assisted rapid palatal expansion by maxillary widening device. Journal of Craniofacial Surgery. 2008;19(3):846-9.	Excluded; no conventional maxillary expansion
386	Tausche E, Hansen L, Hietschold V, Lagravere MO, Harzer W. Three-dimensional evaluation of surgically assisted implant bone-borne rapid maxillary expansion: A pilot study. American Journal of Orthodontics and Dentofacial Orthopedics. 2007;131(4):S92-S9.	Excluded; no conventional maxillary expansion
387	Xi T, Laskowska M, van de Voort N, Ghaeminia H, Pawlak W, Berge S, et al. The effects of surgically assisted rapid maxillary expansion (SARME) on the dental show and chin projection. Journal of Cranio-Maxillofacial Surgery. 2017;45(11):1835-41.	Excluded; no conventional maxillary expansion
388	Hua XM, Xiong H, Han GL, Cheng XR. Correction of a dental arch-width asymmetric discrepancy with a slow maxillary contraction appliance. Am J Orthod Dentofacial Orthop 2012;142(6):842-53.	Excluded; no skeletal-anchored expansion
389	Manni A, Pasini M, Giuca MR, Morganti R, Cozzani M. A retrospective cephalometric study on pharyngeal airway space changes after rapid palatal expansion and Herbst appliance with or without skeletal anchorage. Prog Orthod. 2016;17(1):29.	Excluded; no skeletal-anchored expansion
390	Akin M, Akgul YE, Ileri Z, Basciftci FA. Three-dimensional evaluation of hybrid expander appliances: A pilot study. Angle Orthod. 2016;86(1):81-6.	Excluded; no dental-anchored expansion
391	Asscherickx K, Govaerts E, Aerts J, Vande Vannet B. Maxillary changes with bone-borne surgically assisted rapid palatal expansion: A prospective study. American Journal of Orthodontics and Dentofacial Orthopedics. 2016;149(3):374-83.	Excluded; no dental-anchored expansion
392	Buschang PH, Carrillo R, Rossouw PE. Orthopedic correction of growing hyperdivergent, retrognathic patients with miniscrew implants. J Oral Maxillofac Surg. 2011;69(3):754-62.	Excluded; no dental-anchored expansion
393	Cantarella D, Dominguez-Mompell R, Mallya SM, Moschik C, Pan HC, Miller J, et al. Changes in the midpalatal and pterygopalatine sutures induced by micro-implant-supported skeletal expander, analyzed with a novel 3D method based on CBCT imaging. Prog Orthod. 2017;18(1):34.	Excluded; no dental-anchored expansion
394	Cantarella D, Dominguez-Mompell R, Mallya SM, Moschik C, Pan HC, Miller J, et al. Changes in the midpalatal and pterygopalatine sutures induced by micro-implant-supported skeletal expander, analyzed with a novel 3D method based on CBCT imaging. Progress in Orthodontics. 2017;18(1).	Excluded; no dental-anchored expansion
395	Cantarella D, Dominguez-Mompell R, Moschik C, Mallya SM, Pan HC, Alkahtani MR, et al. Midfacial changes in the coronal plane induced by microimplant-supported skeletal expander, studied with cone-beam computed tomography images. Am J Orthod Dentofacial Orthop. 2018;154(3):337-45.	Excluded; no dental-anchored expansion
396	Carlino F, Villani GP, Berti A, Pantaleo G, Cortese A, Claudio PP. Osteodistraction With Dental Implant-Borne Devices for Bone Regeneration in Atrophied Premaxilla. J Craniofac Surg. 2016;27(8):e776-e9.	Excluded; no dental-anchored expansion
397	Choi SH, Shi KK, Cha JY, Park YC, Lee KJ. Nonsurgical miniscrew-assisted rapid maxillary expansion results in acceptable stability in young adults. Angle Orthod 2016;86(5):713-20.	Excluded; no dental-anchored expansion
398	Choi SH, Shi KK, Cha JY, Park YC, Lee KJ. Nonsurgical miniscrew-assisted rapid maxillary expansion results in acceptable stability in	Excluded; no dental-anchored

	young adults. Angle Orthod. 2016;86(5):713-20.	expansion
399	Clement EA, Krishnaswamy NR. Skeletal and Dentoalveolar Changes after Skeletal Anchorage-assisted Rapid Palatal Expansion in Young Adults: A Cone Beam Computed Tomography Study. Apos Trends in Orthodontics. 2017;7(3):113-9.	Excluded; no dental-anchored expansion
400	Cortese A, Savastano M, Savastano G, Papa F, Howard CM, Claudio PP. Maxillary Constriction Treated by a New Palatal Distractor Device: Surgical and Occlusal Evaluations of 10 Patients. Journal of Craniofacial Surgery. 2010;21(2):339-43.	Excluded; no dental-anchored expansion
401	Deeb W, Hansen L, Hotan T, Hietschold V, Harzer W, Tausche E. Changes in nasal volume after surgically assisted bone-borne rapid maxillary expansion. American Journal of Orthodontics and Dentofacial Orthopedics. 2010;137(6):782-9.	Excluded; no dental-anchored expansion
402	Farhangfar A, Bogowicz P, Heo G, Lagravère MO. Palatal bone resorption in bone-anchored maxillary expander treatment. International Orthodontics. 2012;10(3):274-88.	Excluded; no dental-anchored expansion
403	Hourfar J, Ruff CJ, Wilmes B, Ludwig B, Kanavakis G. Rapid Maxillary Expansion and Upper-Molar Distalization with a Miniscrew-Supported Hybrid Appliance. J Clin Orthod. 2016;50(8):476-84.	Excluded; no dental-anchored expansion
404	Hur JS, Kim HH, Choi JY, Suh SH, Baek SH. Investigation of the effects of miniscrew-assisted rapid palatal expansion on airflow in the upper airway of an adult patient with obstructive sleep apnea syndrome using computational fluid-structure interaction analysis. Korean J Orthod 2017;47(6):353-64.	Excluded; no dental-anchored expansion
405	Iida S, Haraguchi S, Aikawa T, Yashiro K, Okura M, Kogo M. Conventional bone-anchored palatal distractor using an orthodontic palatal expander for the transverse maxillary distraction osteogenesis: technical note. Oral Surg Oral Med Oral Pathol Oral Radiol Endod. 2008;105(2):e8-11.	Excluded; no dental-anchored expansion
406	Kim KB, Helmkamp ME. Miniscrew implant-supported rapid maxillary expansion. J Clin Orthod. 2012;46(10):608-12; quiz 31.	Excluded; no dental-anchored expansion
407	Lee SY, Choi YJ. Skeletal and dentoalveolar changes after miniscrew-assisted rapid palatal expansion in young adults: A cone-beam computed tomography study. Korean J Orthod 2017;47(4):213-4.	Excluded; no dental-anchored expansion
408	Lim HM, Park YC, Lee KJ, Kim KH, Choi YJ. Stability of dental, alveolar, and skeletal changes after miniscrew-assisted rapid palatal expansion. Korean J Orthod 2017;47(5):313-22.	Excluded; no dental-anchored expansion
409	Maino BG, Paoletto E, Lombardo L, Siciiani G. From Planning to Delivery of a Bone-Borne Rapid Maxillary Expander in One Visit. J Clin Orthod. 2017;51(4):198-207.	Excluded; no dental-anchored expansion
410	Ngan P, Nguyen UK, Nguyen T, Tremont T, Martin C. Skeletal, Dentoalveolar, and Periodontal Changes of Skeletally Matured Patients with Maxillary Deficiency Treated with Microimplant-assisted Rapid Palatal Expansion Appliances: A Pilot Study. Apos Trends in Orthodontics. 2018;8(2):71-85.	Excluded; no dental-anchored expansion
411	Ramieri GA, Nasi A, Dell'Acqua A, Verzé L. Facial soft tissue changes after transverse palatal distraction in adult patients. International Journal of Oral and Maxillofacial Surgery. 2008;37(9):810-8.	Excluded; no dental-anchored expansion
412	Seo YJ, Chung KR, Kim SH, Nelson G. Camouflage treatment of skeletal class III malocclusion with asymmetry using a bone-borne rapid maxillary expander. Angle Orthod. 2015;85(2):322-34.	Excluded; no dental-anchored expansion
413	Seo YJ, Lin L, Kim SH, Chung KR, Nelson G. Strategic camouflage treatment of skeletal Class III malocclusion (mandibular prognathism) using bone-borne rapid maxillary expansion and mandibular anterior subapical osteotomy. Am J Orthod Dentofacial Orthop. 2016;149(1):114-26.	Excluded; no dental-anchored expansion
414	Tausche E, Hansen L, Hietschold V, Lagravere MO, Harzer W. Three-dimensional evaluation of surgically assisted implant bone-borne rapid maxillary expansion: a pilot study. Am J Orthod Dentofacial Orthop. 2007;131(4 Suppl):S92-9.	Excluded; no dental-anchored expansion
415	Vassar JW, Karydis A, Trojan T, Fisher J. Dentoskeletal effects of a temporary skeletal anchorage device-supported rapid maxillary expansion appliance (TSADRME): A pilot study. Angle Orthod. 2016;86(2):241-9.	Excluded; no dental-anchored expansion
416	Vassar JW, Karydis A, Trojan T, Fisher J. Dentoskeletal effects of a temporary skeletal anchorage device-supported rapid maxillary expansion appliance (TSADRME): A pilot study. Angle Orthodontist. 2016;86(2):241-9.	Excluded; no dental-anchored expansion
417	Willmann JH, Nienkemper M, Becker K, Ihssen B, Wilmes B, Drescher D. The Hybrid Hyrax Distalizer - Skeletal Anchorage Device for Transversal and Sagittal Corrections in the Maxilla. Informationen Aus Orthodontie Und Kieferorthopaedie. 2016;48(4):228-36.	Excluded; no dental-anchored expansion
418	Wilmes B, Nienkemper M, Drescher D. Application and effectiveness of a mini-implant- and tooth-borne rapid palatal expansion device: the hybrid hyrax. World J Orthod. 2010;11(4):323-30.	Excluded; no dental-anchored expansion
419	Winsauer H, Vlachojannis J, Winsauer C, Ludwig B, Walter A. A bone-borne appliance for rapid maxillary expansion. J Clin Orthod. 2013;47(6):375-81; quiz 88.	Excluded; no dental-anchored expansion
420	Lin L, Ahn HW, Kim SJ, Moon SC, Kim SH, Nelson G. Tooth-borne vs bone-borne rapid maxillary expanders in late adolescence. Angle Orthod. 2015;85(2):253-62.	Excluded; retrospective study
421	Lin L, Ahn HW, Kim SJ, Moon SC, Kim SH, Nelson G. Tooth-borne vs bone-borne rapid maxillary expanders in late adolescence. Angle Orthodontist. 2015;85(2):253-62.	Excluded; retrospective study
422	Yilmaz A, Arman-Ozcirpici A, Erken S, Polat-Ozsoy O. Comparison of short-term effects of mini-implant-supported maxillary expansion appliance with two conventional expansion protocols. Eur J Orthod. 2015;37(5):556-64.	Excluded; retrospective study
423	Yilmaz A, Arman-Ozcirpici A, Erken S, Polat-Ozsoy O. Comparison of short-term effects of mini-implant-supported maxillary expansion appliance with two conventional expansion protocols. European Journal of Orthodontics. 2015;37(5):556-64.	Excluded; retrospective study
424	Mosleh MI, Kaddah MA, Abd ElSayed FA, ElSayed HS. Comparison of transverse changes during maxillary expansion with 4-point bone-borne and tooth-borne maxillary expanders. Am J Orthod Dentofacial Orthop. 2015;148(4):599-607.	Excluded; non-randomized
Inclusion		
425	Bazargani F, Magnuson A, Ludwig B. Effects on nasal airflow and resistance using two different RME appliances: a randomized controlled trial. Eur J Orthod. 2018;40(3):281-4.	Included; randomized
426	Canan S, Senisik NE. Comparison of the treatment effects of different rapid maxillary expansion devices on the maxilla and the mandible. Part 1: Evaluation of dentoalveolar changes. Am J Orthod Dentofacial Orthop. 2017;151(6):1125-38.	Included; randomized
427	Celenk-Koca T, Erdinc AE, Hazar S, Harris L, English JD, Akyalcin S. Evaluation of miniscrew-supported rapid maxillary expansion in adolescents: (A prospective randomized clinical trial). Angle Orthod 2018 [Epub ahead of print]	Included; randomized
428	Feldmann I, Bazargani F. Pain and discomfort during the first week of rapid maxillary expansion (RME) using two different RME appliances: A randomized controlled trial. Angle Orthod 2017;87(3):391-6.	Included; randomized
429	Gunyuz Toklu M, Germec-Cakan D, Tozlu M. Periodontal, dentoalveolar, and skeletal effects of tooth-borne and tooth-bone-borne expansion appliances. Am J Orthod Dentofacial Orthop. 2015;148(1):97-109.	Included; randomized
430	Kabalan O, Gordon J, Heo G, Lagravere MO. Nasal airway changes in bone-borne and tooth-borne rapid maxillary expansion treatments. Int Orthod. 2015;13(1):1-15.	Included; randomized
431	Lagravere MO, Carey J, Heo G, Toogood RW, Major PW. Transverse, vertical, and anteroposterior changes from bone-anchored maxillary expansion vs traditional rapid maxillary expansion: a randomized clinical trial. Am J Orthod Dentofacial Orthop. 2010;137(3):304.e1-12; discussion -5.	Included; randomized
432	Lagravere MO, Gamble J, Major PW, Heo G. Transverse dental changes after tooth-borne and bone-borne maxillary expansion. Int Orthod. 2013;11(1):21-34.	Included; randomized
433	Stepanko LS, Lagravère MO. Sphenoid bone changes in rapid maxillary expansion assessed with cone-beam computed tomography. Korean J Orthod 2016;46(5):269-79.	Included; randomized
434	Forst DD. External Root Resorption Associated with Maxillary Expansion Therapies as Evaluated via Cone Beam Computed Tomography: A Retrospective Randomized Clinical Trial. MSc Thesis, 2015, University of Alberta.	Included; randomized
435	Lagrevere MOV. Analysis of Skeletal and Dental Changes with a Tooth-Borne and a Bone-Borne Maxillary Expansion Appliance assessed through Digital Volumetric Imaging. PhD Thesis, 2009, University of Alberta.	Included; randomized

Appendix 4. Details of the expansion appliances used in the included trials.

Study	Bone anchorage (bone-borne or hybrid)	Tooth anchorage (tooth-borne)
Bazargani 2018	**Hybrid RME**<br>▪ 2 x 1.7 × 8 mm mini-screw implants (Orthoeasy®; Forestadent, Pforzheim, Germany) at the anterior palate<br>▪ 1st molar bands soldered to the expander	**Tooth-borne RME**<br>▪ 1st molar bands soldered to the expander; extension arms at the 1st / 2nd primary molars
Canan 2017	**Bone-borne RME**<br>▪ 4 x 1.8 × 9 mm pre-drilling mini-screw implants (Yesanchor; Seoul, Korea) at the palatal sides<br><br>**Hybrid RME**<br>▪ 2 x 1.8 × 9 mm pre-drilling mini-screw implants (Yesanchor; Seoul, Korea) at the posterior palate<br>▪ 1st molar bands soldered to the expander	**Tooth-borne RME**<br>▪ 1st premolar & 1st molar bands connected and soldered to the expander
Celenk-Koca 2018	**Bone-borne RME**<br>▪ 4 x 1.8 × 9 mm pre-drilling mini-screw implants (Orlus; Ortholution Co, Seoul, Korea) at the palatal sides	**Tooth-borne RME**<br>▪ Cast expander on the 1st premolar, 2nd premolar, & 1st molar with extension arm for the 2nd molar
Feldmann 2017	**Hybrid RME**<br>▪ 2 x 1.7 × 8 mm mini-screw implants (Orthoeasy®; Forestadent, Pforzheim, Germany) at the anterior palate<br>▪ 1st molar bands soldered to the expander	**Tooth-borne RME**<br>▪ 1st molar bands soldered to the expander; extension arms at the 1st / 2nd primary molars
Gunyuz Toklu 2015	**Hybrid RME**<br>▪ 2 x 1.8 × 9 mm mini-screw implants (Total Anchor; Trimed, Ankara, Turkey) at the anterior palate<br>▪ 1st molar bands soldered to the expander	**Tooth-borne RME**<br>▪ 1st premolar & 1st molar bands connected and soldered to the expander
Lagravere 2009$_{collated}$†	**Hybrid RME**<br>▪ 2 x onplants and 2 x 1.5 × 12 mm mini-screw implants (Straumann GBR-System; Andover, Mass) at the anterior palate	**Tooth-borne RME**<br>▪ 1st premolar & 1st molar bands connected and soldered to the expander

RME, rapid maxillary expansion

Appendix 5. Risk of bias assessment of included randomized trials.

**Study**	Sequence generation	Allocation concealment	Blinding of participants/ personnel	Blinding of outcome assessors	Incomplete outcome data	Selective outcome reporting	Other sources of bias
Bazargani 2018	Low risk - "Participants were randomly allocated in blocks of different sizes, using the concealed allocation principle in a 1:1 ratio, to two groups, a TB group and a TBB group. The randomization procedure was as follows: a computer-generated randomization list…"	Low risk – central allocation: "…and stored with a research secretary at the Postgraduate Dental Education Centre. Each time a patient gave his/her consent, the secretary was contacted by e-mail to provide the information about which type of expander the patient should receive."	Low risk – patients and treatment providers were not blinded, but treatment providers could have been. However, the outcome is objective and has been assessed blindly.	Low risk – "The intermolar measurements were blinded; the examiner was unaware of which treatment the patients had received or which models were taken at pre- and post-expansion.<br>The care providers at the ENT unit who conducted all the rhinomanometry examinations were blinded to which group the<br>patients were allocated to".	Unclear – double attrition rate for the more invasive group (33%) compared to the conventional group (16%); although imputation methods were used to assess the effect of attrition on the results, higher attrition might indicate lower tolerability of the intervention.	Unclear – no obvious selective reporting; however, only dental arch width is assessed and no dental inclination or skeletal width is assessed; finally, it is difficult to judge however whether selective reporting is a problem, as no protocol exists.	Low risk – no other sources of bias; intervention is dependent on patient compliance (which is not reported), but as treatment end was based on objective effects (expansion), this was considered irrelevant.
Canan 2017	Unclear - "Three study groups were designated with stratified randomization (strata, sex)…"	Unclear – No mention throughout the paper.	Unclear - Blinding of participants and personnel impossible; outcome is objective but it is unclear if assessed blindly.	High risk – No mention of blinding throughout the paper; blinding however is possible.	Low risk – 6 drop-outs (11%) after randomization (5 after treatment administration); although reasons for drop-outs might be related to treatment tolerability/acceptance, drop-out rate is relatively low.	Unclear – no obvious selective reporting; however, it is difficult to judge however whether selective reporting is a problem, as no protocol exists.	Low risk – no other sources of bias; intervention is dependent on patient compliance (which is not reported), but as treatment end was based on objective effects (expansion) and expansion duration is reported, this was considered irrelevant.
Celenk-Koca 2018	Low risk – "Patients were randomly assigned to one of the two treatment groups via a block randomization procedure with a block size of four, using a computer-generated list of random numbers".	Low risk – "The allocation sequence was concealed from the orthodontist, researchers, and the patients. When a patient was deemed as eligible for enrollment, the patient was assigned to a treatment group using opaque and sealed envelopes containing the allocation number."	Low risk - Blinding of participants and personnel impossible; outcome is objective and has been assessed and analyzed blindly.	Low risk – "Since it was impossible to blind the patient and orthodontist to the treatment groups, the researcher who traced the cone-beam computed tomography (CBCT) images and the statistician who evaluated the data were blinded".	Low risk – no drop outs.	Low risk – no obvious selective reporting; although it is difficult to judge however whether selective reporting is a problem, as no protocol exists, all possible dental/skeletal outcomes that could be measured by CBCT are reported.	Low risk – no other sources of bias; intervention is dependent on patient compliance (which is not reported), but as treatment end was based on objective effects (expansion), this was considered irrelevant.
Feldmann 2017	Low risk - "…a computer-generated randomization list was created using SPSS software (version 17.0; SPSS, Chicago, Ill) and stored with a research secretary at the Postgraduate Dental Education Centre"	Low risk – central allocation: "Each time a patient gave his/her consent, the secretary was contacted by e-mail and the information about which type of expander the patient should receive was acquired".	Low risk - Blinding of participants and personnel impossible; outcome is subjective but was assessed blindly.	Low risk – "The questionnaires were analyzed by one of the coauthors, who was blinded to the study and performed no orthodontic treatment on the patients."	Low risk – low drop-out rate (7%) which is transparently reported and balanced and the post-drop-out similarity is assessed.	Low risk – no obvious selective reporting; although it is difficult to judge however whether selective reporting is a problem, as no protocol exists, all possible patient-reported outcomes that could be measured within a trial with so short span (1 week) are reported.	Unclear –intervention (and probably outcome) is dependent on patient compliance (which is not reported) and no information given about patient compliance with given instructions.
Gunyuz Toklu 2015	High risk – randomization is unclear, but resembles more an alteration scheme: "According to the order of referral with a randomization ratio of 1:1, they were randomly allocated to 2 groups by an orthodontist (D.G.-C.) who did not know in advance which treatment the next patient would get."	Low risk – central allocation; see sequence generation text.	Unclear - Blinding of participants and personnel impossible; outcome is objective but it is unclear if assessed blindly.	High risk – No mention of blinding of outcome assessment, even though this is possible. Only measurement of the statistician, which is not equally important: "All measurements were made by the same researcher (M.G.T.). Blinding was used at the analysis level."	Low risk – Low drop-out rate (4%): "One patient who lost the palatal miniscrews 2 days after insertion of the expander because of consuming hard foods was excluded from the study."	Unclear – no obvious selective reporting; however, it is difficult to judge however whether selective reporting is a problem, as no protocol exists.	Low risk – no other sources of bias; intervention is dependent on patient compliance (which is not reported), but as treatment end was based on objective effects (expansion) and expansion duration is reported, this was considered irrelevant.
Lagravere 2009$_{collated}$[†]	Low risk - "…the subjects were randomized into the groups by using a random numbers generated list."	Unclear – No mention throughout the paper.	Unclear – Blinding of participants and personnel impossible; outcome is objective but it is unclear if assessed blindly.	High risk – No mention of blinding throughout the paper; blinding however is possible.	Low risk - No drop-outs or patient losses are reported.	Unclear – no obvious selective reporting; however, it is difficult to judge however whether selective reporting is a problem, as no protocol exists.	Low risk – no other sources of bias; intervention is dependent on patient compliance (which is not reported), but as treatment end was based on objective effects (expansion), this was considered irrelevant.

Appendix 6a. List of included trials comparing bone-borne with tooth-borne rapid maxillary expansion.

Outcome category	Trial	Outcome	Timing	MD	95% CI	P	Clinically relevant$^{\$}$
Skeletal$_{maxilla}$	Lagravere 2009	External maxilla width at 1st molar	Pst-Exp	-0.53	-1.48, 0.42	0.27	-
Skeletal$_{maxilla}$	Lagravere 2009	External maxilla width at 1st premolar	Pst-Exp	-0.26	-1.41, 0.89	0.66	-
Skeletal$_{maxilla}$	Lagravere 2009	External maxilla width at pterygoid	Pst-Exp	-0.39	-1.71, 0.93	0.56	-
Dental$_{position}$	Canan 2017	Intercanine width (cusp)	Pst-Exp	-0.70	-0.97, -0.43	<0.001	Yes
Dental$_{position}$	Lagravere 2009	Inter-central-incisor width (apex)	Pst-Exp	-0.56	-1.65, 0.53	0.32	-
Dental$_{position}$	Lagravere 2009	Inter-central-incisor width (pulp chamber)	Pst-Exp	-0.87	-1.77, 0.03	0.06	-
Dental$_{position}$	Lagravere 2009	Intermolar width (apex)	Pst-Exp	0.08	-0.82, 0.98	0.86	-
Dental$_{position}$	Canan 2017	Intermolar width (cusp)	Pst-Exp	-0.09	-0.34, 0.16	0.48	-
Dental$_{position}$	Lagravere 2009	Intermolar width (pulp chamber)	Pst-Exp	-0.15	-1.30, 1.00	0.80	-
Dental$_{position}$	Lagravere 2009	Inter-1st-premolar width (apex)	Pst-Exp	-0.52	-1.53, 0.49	0.31	-
Dental$_{position}$	Canan 2017	Inter-1st-premolar width (cusp)	Pst-Exp	0.23	-0.15, 0.61	0.23	-
Dental$_{position}$	Lagravere 2009	Inter-1st-premolar width (pulp chamber)	Pst-Exp	-1.80	-2.92, -0.68	0.002	No
Dental$_{inclination}$	Canan 2017	Inclination 1st molar (left)	Pst-Exp	-5.39	-8.04, -2.74	<0.001	Yes
Dental$_{inclination}$	Lagravere 2009	Inclination 1st molar (left)	Pst-Exp	-0.35	-3.40, 2.70	0.82	-
Dental$_{inclination}$	Canan 2017	Inclination 1st molar (right)	Pst-Exp	-2.12	-5.46, 1.22	0.21	-
Dental$_{inclination}$	Lagravere 2009	Inclination 1st molar (right)	Pst-Exp	-0.76	-4.27, 2.75	0.67	-
Dental$_{inclination}$	Canan 2017	Inclination 1st premolar (left)	Pst-Exp	-4.03	-6.79, -1.27	0.004	No
Dental$_{inclination}$	Lagravere 2009	Inclination 1st premolar (left)	Pst-Exp	-1.25	-3.33, 0.83	0.24	-
Dental$_{inclination}$	Canan 2017	Inclination 1st premolar (right)	Pst-Exp	-4.28	-6.92, -1.64	0.001	Yes
Dental$_{inclination}$	Lagravere 2009	Inclination 1st premolar (right)	Pst-Exp	-3.79	-6.60, -0.98	0.008	No
Nasal cavity	Lagravere 2009	Nasal cavity width at orbita	Pst-Exp	0.24	-0.13, 0.61	0.20	-
Cranial$_{vertical}$	Lagravere 2009	Height orbita-incisor (left)	Pst-Exp	-0.50	-1.79, 0.79	0.45	-
Cranial$_{vertical}$	Lagravere 2009	Height orbita-incisor (right)	Pst-Exp	-1.40	-2.48, -0.32	0.01	No
Cranial$_{vertical}$	Lagravere 2009	Height orbita-1st molar (left)	Pst-Exp	0.02	-1.53, 1.57	0.98	-
Cranial$_{vertical}$	Lagravere 2009	Height orbita-1st molar (right)	Pst-Exp	-0.38	-1.36, 0.60	0.45	-
Cranial$_{vertical}$	Lagravere 2009	Height orbita-menton (left)	Pst-Exp	-0.67	-2.58, 1.24	0.49	-
Cranial$_{vertical}$	Lagravere 2009	Height orbita-menton (right)	Pst-Exp	-0.80	-1.68, 1.08	0.40	-
Mandible	Lagravere 2009	Lower intermolar width (pulpal chamber)	Pst-Exp	-0.56	-1.28, 0.16	0.13	-
Mandible	Lagravere 2009	Mandible width at foramen	Pst-Exp	0.30	-0.07, 0.67	0.11	-
Skeletal$_{maxilla}$	Celenk-Koca 2018	Buccal bone thickness at 1st molar	Reten	0.14	0.04, 0.24	0.005	No
Skeletal$_{maxilla}$	Celenk-Koca 2018	Buccal bone thickness at 1st premolar	Reten	0.25	0.13, 0.37	<0.001	Yes
Skeletal$_{maxilla}$	Celenk-Koca 2018	Incisal foramen width	Reten	1.80	1.27, 2.33	<0.001	Yes
Skeletal$_{maxilla}$	Lagravere 2009	External maxilla width at 1st molar	Reten	-0.70	-1.83, 0.43	0.22	-
Skeletal$_{maxilla}$	Lagravere 2009	External maxilla width at 1st premolar	Reten	-1.01	-2.23, 0.21	0.22	-
Skeletal$_{maxilla}$	Lagravere 2009	External maxilla width at pterygoid	Reten	-0.33	-1.71, 1.05	0.11	-
Skeletal$_{maxilla}$	Celenk-Koca 2018	Parallel suture opening	Reten	*2.50	*0.55, 11.41	0.24	-
Skeletal$_{maxilla}$	Celenk-Koca 2018	Suture width at 1st molar	Reten	2.00	1.40, 2.60	<0.001	Yes
Skeletal$_{maxilla}$	Celenk-Koca 2018	Suture width at 1st premolar	Reten	2.30	1.69, 2.91	<0.001	Yes
Dental$_{position}$	Lagravere 2009	Inter-central-incisor width (apex)	Reten	-0.33	-1.26, 0.60	0.49	-
Dental$_{position}$	Lagravere 2009	Inter-central-incisor width (pulpal chamber)	Reten	-0.11	-0.67, 0.45	0.70	-
Dental$_{position}$	Canan 2017	Intercanine width (cusp)	Reten	-0.51	-0.95, -0.07	0.02	Yes
Dental$_{position}$	Lagravere 2009	Intermolar width (apex)	Reten	-0.71	-1.87, 0.45	0.23	-
Dental$_{position}$	Canan 2017	Intermolar width (cusp)	Reten	0.15	-0.36, 0.66	0.57	-
Dental$_{position}$	Celenk-Koca 2018	Intermolar width (palatal crown middle)	Reten	0.30	-0.64, 1.24	0.53	-
Dental$_{position}$	Lagravere 2009	Intermolar width (pulpal chamber)	Reten	-0.08	-1.16, 1.00	0.89	-
Dental$_{position}$	Lagravere 2009	Inter-1st-premolar width (apex)	Reten	-1.81	-3.07, -0.55	0.005	No
Dental$_{position}$	Canan 2017	Inter-1st-premolar width (cusp)	Reten	-0.39	-1.07, 0.29	0.26	-
Dental$_{position}$	Celenk-Koca 2018	Inter-1st-premolar width (palatal	Reten	0.60	-1.18, 2.38	0.51	-

		crown middle)					
Dental$_{position}$	Lagravere 2009	Inter-1st-premolar width (pulpal chamber)	Reten	-1.76	-2.66, -0.86	<0.001	Yes
Dental$_{inclination}$	Celenk-Koca 2018	Inclination 1st molar (right-left average)	Reten	-5.20	-6.95, -3.45	<0.001	Yes
Dental$_{inclination}$	Canan 2017	Inclination 1st molar (left)	Reten	-5.83	-9.98, -1.68	0.006	Yes
Dental$_{inclination}$	Lagravere 2009	Inclination 1st molar (left)	Reten	1.92	-1.74, 5.58	0.30	-
Dental$_{inclination}$	Canan 2017	Inclination 1st molar (right)	Reten	-2.38	-6.30, 1.54	0.23	-
Dental$_{inclination}$	Lagravere 2009	Inclination 1st molar (right)	Reten	1.44	-1.28, 4.16	0.30	-
Dental$_{inclination}$	Celenk-Koca 2018	Inclination 1st premolar (right-left average)	Reten	-5.10	-6.78, -3.42	<0.001	Yes
Dental$_{inclination}$	Canan 2017	Inclination 1st premolar (left)	Reten	-6.21	-10.13, -2.30	0.002	No
Dental$_{inclination}$	Lagravere 2009	Inclination 1st premolar (left)	Reten	1.09	-1.25, 3.43	0.36	-
Dental$_{inclination}$	Canan 2017	Inclination 1st premolar (right)	Reten	-1.39	-5.17, 2.39	0.47	-
Dental$_{inclination}$	Lagravere 2009	Inclination 1st premolar (right)	Reten	-0.43	-3.24, 2.38	0.76	-
Nasal cavity	Lagravere 2009	Nasal cavity width (cross-section 1; left)	Reten	-0.02	-0.06, 0.02	0.36	-
Nasal cavity	Lagravere 2009	Nasal cavity width (cross-section 1; right)	Reten	0.02	-0.04,0.08	0.48	-
Nasal cavity	Lagravere 2009	Nasal cavity width (cross-section 2; left)	Reten	1.04	-0.87, 2.95	0.29	-
Nasal cavity	Lagravere 2009	Nasal cavity width (cross-section 2; right)	Reten	0.02	-0.03, 0.07	0.39	-
Nasal cavity	Lagravere 2009	Nasal cavity volume 1 (left)	Reten	0.42	-0.25, 1.09	0.22	-
Nasal cavity	Lagravere 2009	Nasal cavity volume 1 (right)	Reten	0.39	-0.31, 1.09	0.27	-
Nasal cavity	Lagravere 2009	Nasal cavity volume 2 (left)	Reten	0.15	-0.54, 0.84	0.67	-
Nasal cavity	Lagravere 2009	Nasal cavity volume 2 (right)	Reten	0.68	-0.91, 2.27	0.40	-
Nasal cavity	Celenk-Koca 2018	Nasal cavity width at 1st molar	Reten	1.70	0.81, 2.59	<0.001	Yes
Nasal cavity	Lagravere 2009	Nasal cavity width at orbita	Reten	0.17	-0.24, 0.58	0.42	-
Nasal cavity	Celenk-Koca 2018	Nasal cavity width at 1st premolar	Reten	1.00	-0.09, 2.09	0.07	-
Cranial$_{vertical}$	Lagravere 2009	Height orbita-incisor (left)	Reten	-0.18	-0.96, 0.60	0.65	-
Cranial$_{vertical}$	Lagravere 2009	Height orbita-incisor (right)	Reten	-0.51	-1.17, 0.15	0.13	-
Cranial$_{vertical}$	Lagravere 2009	Height orbita-1st molar (left)	Reten	0.40	-0.32, 1.12	0.27	-
Cranial$_{vertical}$	Lagravere 2009	Height orbita-1st molar (right)	Reten	0.25	-0.51, 1.01	0.52	-
Cranial$_{vertical}$	Lagravere 2009	Height orbita-menton (left)	Reten	0.03	-0.91, 0.97	0.95	-
Cranial$_{vertical}$	Lagravere 2009	Height orbita-menton (right)	Reten	0.05	-1.01, 1.11	0.93	-
Mandible	Lagravere 2009	Lower intermolar width (pulpal chamber)	Reten	-0.20	-0.72, 0.32	0.45	-
Mandible	Lagravere 2009	Mandible width at foramen	Reten	0.20	-0.19, 0.59	0.31	-
Root resorption	Celenk-Koca 2018	Root resorption linear (1st molar mesiobuccal root)	Reten	0.06	-0.04, 0.16	0.23	-
Root resorption	Celenk-Koca 2018	Root resorption linear (1st molar mesiodistal root)	Reten	-0.07	-0.19, 0.05	0.27	-
Root resorption	Celenk-Koca 2018	Root resorption linear (1st molar palatal root)	Reten	0.07	-0.03, 0.17	0.16	-
Root resorption	Celenk-Koca 2018	Root resorption linear (1st premolar buccal root)	Reten	0.13	0.01, 0.25	0.04	No
Root resorption	Celenk-Koca 2018	Root resorption linear (1st premolar palatal root)	Reten	0.09	-0.05, 0.23	0.20	-
Root resorption	Lagravere 2009	Root resorption volume (1st molar)	Reten	-17.82	-46.01, 10.37	0.22	-
Root resorption	Lagravere 2009	Root resorption % volume (1st molar)	Reten	-1.51	-3.91, 0.89	0.22	-

CI, confidence interval; MD, mean difference; Pst-Exp, post expansion; Reten, post retention period (at least 3 months).
* pertains to risks and not mean difference.
Judged arbitrarily as mean difference being equal or greater to one standard deviation of the control group.

Appendix 6b. List of included trials comparing hybrid (tooth-bone-borne) with tooth-borne rapid maxillary expansion.

Outcome category	Trial	Outcome	Timing	MD	95% CI	P	Clinically relevant[$]
Pain	Feldmann 2017	Analgesic use on day 4	Mid-Exp	*0.78	*0.34, 1.76	0.55	-
Pain	Feldmann 2017	Analgesic use on day 4	Mid-Exp	*0.40	*0.09, 1.87	0.25	-
Dental$_{position}$	Canan 2017	Intercanine width (cusp)	Pst-Exp	-0.68	-0.94, -0.42	<0.001	Yes
Dental$_{position}$	Canan 2017	Intermolar width (cusp)	Pst-Exp	-0.06	-0.36, 0.24	0.69	-
Dental$_{position}$	Canan 2017	Inter-1st-premolar width (cusp)	Pst-Exp	0.14	-0.28, 0.56	0.52	-
Dental$_{inclination}$	Canan 2017	Inclination 1st molar (left)	Pst-Exp	-1.45	-4.03, 1.13	0.27	-
Dental$_{inclination}$	Canan 2017	Inclination 1st molar (right)	Pst-Exp	3.20	-0.62, 7.02	0.10	-
Dental$_{inclination}$	Canan 2017	Inclination 1st premolar (left)	Pst-Exp	-3.31	-6.85, 0.23	0.07	-
Dental$_{inclination}$	Canan 2017	Inclination 1st premolar (right)	Pst-Exp	-1.16	-4.32, 2.00	0.47	-
Nasal cavity	Bazargani 2018	Nasal airflow	Pst-Exp	57.70	1.44, 113.96	0.04	No
Nasal cavity	Bazargani 2018	Nasal resistance	Pst-Exp	-0.23	-0.43, -0.04	0.02	No
Skeletal$_{maxilla}$	Gunyuz Toklu 2015	Buccal bone thickness at canine (left)	Reten	-0.25	-0.63, 0.13	0.20	-
Skeletal$_{maxilla}$	Gunyuz Toklu 2015	Buccal bone thickness at canine (right)	Reten	0.00	-0.28, 0.28	1.00	-
Skeletal$_{maxilla}$	Gunyuz Toklu 2015	Buccal bone thickness at 1st molar (averaged)	Reten	-0.15	-0.66, 0.36	0.56	-
Skeletal$_{maxilla}$	Gunyuz Toklu 2015	Buccal bone thickness at 1st molar (distobuccal root; left)	Reten	-0.21	-0.65, 0.23	0.35	-
Skeletal$_{maxilla}$	Gunyuz Toklu 2015	Buccal bone thickness at 1st molar (distobuccal root; right)	Reten	-0.13	-0.77, 0.51	0.69	-
Skeletal$_{maxilla}$	Gunyuz Toklu 2015	Buccal bone thickness at 1st molar (mesiobuccal root; left)	Reten	-0.11	-0.64, 0.42	0.69	-
Skeletal$_{maxilla}$	Gunyuz Toklu 2015	Buccal bone thickness at 1st molar (mesiobuccal root; right)	Reten	-0.15	-0.58, 0.28	0.49	-
Skeletal$_{maxilla}$	Gunyuz Toklu 2015	Buccal bone thickness at 1st premolar (averaged)	Reten	0.63	0.10, 1.16	0.02	Yes
Skeletal$_{maxilla}$	Gunyuz Toklu 2015	Buccal bone thickness at 1st premolar (left)	Reten	0.79	0.30, 1.28	0.001	Yes
Skeletal$_{maxilla}$	Gunyuz Toklu 2015	Buccal bone thickness at 1st premolar (right)	Reten	0.46	-0.13, 1.05	0.13	-
Skeletal$_{maxilla}$	Gunyuz Toklu 2015	Buccal bone thickness at 2nd premolar (left)	Reten	0.15	-0.32, 0.62	0.53	-
Skeletal$_{maxilla}$	Gunyuz Toklu 2015	Buccal bone thickness at 2nd premolar (right)	Reten	-0.11	-0.42, 0.20	0.49	-
Skeletal$_{maxilla}$	Gunyuz Toklu 2015	Buccal bone thickness at canine (left)	Reten	-0.31	-0.96, 0.34	0.35	-
Skeletal$_{maxilla}$	Gunyuz Toklu 2015	Buccal bone thickness at canine (right)	Reten	-0.03	-0.72, 0.66	0.93	-
Skeletal$_{maxilla}$	Gunyuz Toklu 2015	Palatal bone thickness at 1st molar (left)	Reten	0.04	-0.61, 0.69	0.90	-
Skeletal$_{maxilla}$	Gunyuz Toklu 2015	Palatal bone thickness at 1st molar (right)	Reten	-0.43	-0.95, 0.09	0.10	-
Skeletal$_{maxilla}$	Gunyuz Toklu 2015	Palatal bone thickness at 1st premolar (left)	Reten	-1.62	-2.21, -1.03	<0.001	Yes
Skeletal$_{maxilla}$	Gunyuz Toklu 2015	Palatal bone thickness at 1st premolar (right)	Reten	-0.61	-1.55, 0.33	0.20	-
Skeletal$_{maxilla}$	Gunyuz Toklu 2015	Palatal bone thickness at 2nd premolar (left)	Reten	-0.17	-0.67, 0.33	0.51	-
Skeletal$_{maxilla}$	Gunyuz Toklu 2015	Palatal bone thickness at 2nd premolar (right)	Reten	-0.66	-1.25, -0.07	0.03	No
Skeletal$_{maxilla}$	Gunyuz Toklu 2015	External maxilla width at 1st molar	Reten	0.64	-1.41 2.69	0.54	-
Skeletal$_{maxilla}$	Gunyuz Toklu 2015	Palatal maxilla width at 1st molar	Reten	-0.47	-2.29, 1.35	0.61	-
Skeletal$_{maxilla}$	Gunyuz Toklu 2015	External maxilla width at pterygoid	Reten	0.14	-1.09, 1.37	0.82	-
Dental$_{position}$	Gunyuz Toklu 2015	Intercanine width (apex)	Reten	1.69	-0.71, 4.09	0.17	-
Dental$_{position}$	Canan 2017	Intercanine width (cusp)	Reten	-0.01	-0.37, 0.35	0.96	-
Dental$_{position}$	Gunyuz Toklu 2015	Intercanine width (cusp)	Reten	-0.95	-2.39, 0.49	0.19	-
Dental$_{position}$	Gunyuz Toklu 2015	Intermolar width (apex)	Reten	-1.69	-4.39, 1.01	0.22	-
Dental$_{position}$	Canan 2017	Intermolar width (cusp)	Reten	0.14	-0.46, 0.74	0.65	-
Dental$_{position}$	Gunyuz Toklu 2015	Intermolar width (mesiobuccal cusp)	Reten	0.57	-1.38, 2.52	0.57	-

Dental$_{position}$	Gunyuz Toklu 2015	Intermolar width (averaged cusp)	Reten	0.54	-1.38, 2.46	0.58	-
Dental$_{position}$	Gunyuz Toklu 2015	Intermolar width (distobuccal cusp)	Reten	0.52	-1.37, 2.41	0.59	-
Dental$_{position}$	Gunyuz Toklu 2015	Inter-1st-premolar width (apex)	Reten	-3.06	-5.73, -0.39	0.03	No
Dental$_{position}$	Canan 2017	Inter-1st-premolar width (cusp)	Reten	0.00	-0.69, 0.69	1.00	-
Dental$_{position}$	Gunyuz Toklu 2015	Inter-1st-premolar width (buccal cusp)	Reten	-4.33	-7.04, -1.62	0.002	Yes
Dental$_{position}$	Gunyuz Toklu 2015	Inter-1st-premolar width (palatal cusp)	Reten	-5.29	-7.81, -2.77	<0.001	Yes
Dental$_{position}$	Gunyuz Toklu 2015	Inter-2nd-premolar width (apex)	Reten	-0.38	-3.00, 2.24	0.78	-
Dental$_{position}$	Gunyuz Toklu 2015	Inter-2nd-premolar width (buccal cusp)	Reten	-3.33	-6.15, -0.51	0.02	Yes
Dental$_{position}$	Gunyuz Toklu 2015	Inter-2nd-premolar width (palatal cusp)	Reten	-3.48	-6.38, -0.58	0.02	No
Bone$_{inclination}$	Gunyuz Toklu 2015	Alveolar inclination at 1st molar (left)	Reten	-1.02	-5.16, 3.12	0.63	-
Bone$_{inclination}$	Gunyuz Toklu 2015	Alveolar inclination at 1st molar (right)	Reten	-0.35	-4.57, 3.87	0.87	-
Bone$_{inclination}$	Gunyuz Toklu 2015	Alveolar inclination at 1st premolar (left)	Reten	-1.48	-5.00, 2.04	0.41	-
Bone$_{inclination}$	Gunyuz Toklu 2015	Alveolar inclination at 1st premolar (right)	Reten	-1.04	-6.74, 4.66	0.72	-
Dental$_{inclination}$	Gunyuz Toklu 2015	Absolute inclination 1st molar (left)	Reten	0.95	-2.80, 4.70	0.62	-
Dental$_{inclination}$	Gunyuz Toklu 2015	Absolute inclination 1st molar (right)	Reten	-3.99	-8.75, 0.77	0.10	-
Dental$_{inclination}$	Gunyuz Toklu 2015	Absolute inclination 1st premolar (left)	Reten	-1.30	-3.55, 0.95	0.26	-
Dental$_{inclination}$	Gunyuz Toklu 2015	Absolute inclination 1st premolar (right)	Reten	-0.14	-5.60, 5.32	0.96	-
Dental$_{inclination}$	Gunyuz Toklu 2015	Inclination canine (left)	Reten	-3.58	-8.51, 1.35	0.15	-
Dental$_{inclination}$	Gunyuz Toklu 2015	Inclination canine (right)	Reten	0.48	-3.72, 4.68	0.82	-
Dental$_{inclination}$	Canan 2017	Inclination 1st molar (left)	Reten	-2.41	-5.63, 0.81	0.14	-
Dental$_{inclination}$	Gunyuz Toklu 2015	Inclination 1st molar (left)	Reten	-0.07	-3.42, 3.28	0.97	-
Dental$_{inclination}$	Canan 2017	Inclination 1st molar (right)	Reten	1.64	2.63, 5.91	0.45	-
Dental$_{inclination}$	Gunyuz Toklu 2015	Inclination 1st molar (right)	Reten	-4.34	-9.71, 1.03	0.11	-
Dental$_{inclination}$	Canan 2017	Inclination 1st premolar (left)	Reten	-6.09	-10.17, -2.01	0.003	No
Dental$_{inclination}$	Gunyuz Toklu 2015	Inclination 1st premolar (left)	Reten	-2.78	-4.96, -0.61	0.01	No
Dental$_{inclination}$	Canan 2017	Inclination 1st premolar (right)	Reten	-0.06	-4.16, 4.04	0.98	-
Dental$_{inclination}$	Gunyuz Toklu 2015	Inclination 1st premolar (right)	Reten	-1.17	-4.11, 1.77	0.44	-
Dental$_{inclination}$	Gunyuz Toklu 2015	Inclination 2nd premolar (left)	Reten	-1.48	-6.39, 3.43	0.55	-
Dental$_{inclination}$	Gunyuz Toklu 2015	Inclination 2nd premolar (right)	Reten	1.41	-2.04, 4.86	0.42	-
Nasal cavity	Gunyuz Toklu 2015	Nasal cavity width	Reten	0.08	-1.35, 1.51	0.91	-

CI, confidence interval; MD, mean difference; Pst-Exp, post expansion; Reten, post retention period (at least 3 months).

* pertains to risks and not mean difference.

Judged arbitrarily as mean difference being equal or greater to one standard deviation of the control group.
